# Comparative phenotype of circulating versus tissue immune cells in human lung and blood compartments during health and disease

**DOI:** 10.1093/discim/kyad009

**Published:** 2023-07-19

**Authors:** Stefano A P Colombo, Sheila L Brown, Matthew R Hepworth, Jenny Hankinson, Felice Granato, Semra J Kitchen, Tracy Hussell, Angela Simpson, Peter C Cook, Andrew S MacDonald

**Affiliations:** Lydia Becker Institute of Immunology and Inflammation, Faculty of Biology, Medicine, and Health, The University of Manchester, Manchester, UK; Lydia Becker Institute of Immunology and Inflammation, Faculty of Biology, Medicine, and Health, The University of Manchester, Manchester, UK; Lydia Becker Institute of Immunology and Inflammation, Faculty of Biology, Medicine, and Health, The University of Manchester, Manchester, UK; Institute of Translational Genomics, Helmholtz Zentrum München—German Research Center for Environmental Health, Neuherberg, Germany; Department of Cardiothoracic Surgery, Wythenshawe Hospital, Manchester University NHS Foundation Trust, Manchester, UK; GSK, Medicines Research Centre, Gunnels Wood Road, Stevenage, Hertfordshire, UK; Lydia Becker Institute of Immunology and Inflammation, Faculty of Biology, Medicine, and Health, The University of Manchester, Manchester, UK; Lydia Becker Institute of Immunology and Inflammation, Faculty of Biology, Medicine, and Health, The University of Manchester, Manchester, UK; Lydia Becker Institute of Immunology and Inflammation, Faculty of Biology, Medicine, and Health, The University of Manchester, Manchester, UK; MRC Centre for Medical Mycology, University of Exeter, Geoffrey Pope Building, Stocker Road, Exeter, UK; Lydia Becker Institute of Immunology and Inflammation, Faculty of Biology, Medicine, and Health, The University of Manchester, Manchester, UK

**Keywords:** mucosal immunology, T cells, dendritic cells, asthma, COPD

## Abstract

The lung is a dynamic mucosal surface constantly exposed to a variety of immunological challenges including harmless environmental antigens, pollutants, and potentially invasive microorganisms. Dysregulation of the immune system at this crucial site is associated with a range of chronic inflammatory conditions including asthma and Chronic Pulmonary Obstructive Disease (COPD). However, due to its relative inaccessibility, our fundamental understanding of the human lung immune compartment is limited. To address this, we performed flow cytometric immune phenotyping of human lung tissue and matched blood samples that were isolated from 115 donors undergoing lung tissue resection. We provide detailed characterization of the lung mononuclear phagocyte and T cell compartments, demonstrating clear phenotypic differences between lung tissue cells and those in peripheral circulation. Additionally, we show that CD103 expression demarcates pulmonary T cells that have undergone recent TCR and IL-7R signalling. Unexpectedly, we discovered that the immune landscape from asthmatic or COPD donors was broadly comparable to controls. Our data provide a much-needed expansion of our understanding of the pulmonary immune compartment in both health and disease.

## Graphical Abstract

**Graphical Abstract:**
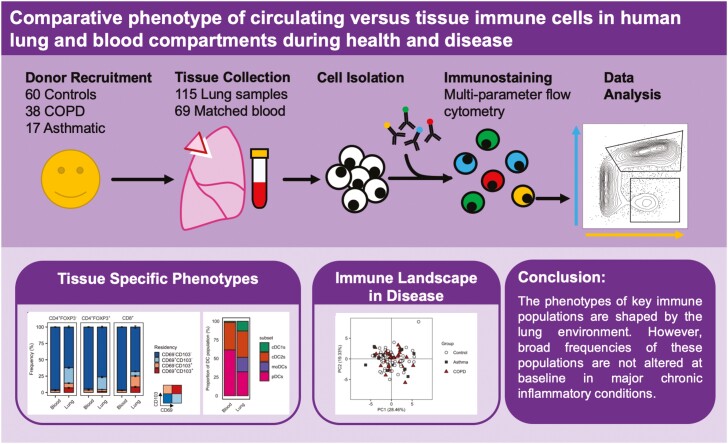

## Introduction

CD45^+^ hematopoietic cells comprise a significant portion of cellular networks within any given organ and provide signals to non-hematopoietic cells to determine tissue function and health. This includes resident innate immune cells surveying the environment for foreign antigen and contributing to tissue homeostasis [1–3], tissue-resident memory cells awaiting re-exposure to their cognate antigen [4, 5], and non-resident cells trafficking through the local vasculature. In the context of immune responses, interaction of resident and non-resident CD45^+^ cells with local tissue cells and the extracellular matrix environment in which they are found is fundamental to the induction, regulation, and resolution of such responses [6, 7]. Consequently, immune responses are highly contextual to tissue environments in which they occur [8].

In humans, sample access for studying tissue-specific immunity is challenging, with our understanding of fundamental human cellular immunity largely derived from peripheral blood cells. While this can be informative [9, 10], studies that have drawn comparisons between immune populations isolated from specific tissues, such as the liver and brain, and those from peripheral blood find clear phenotypic differences between these sites [11, 12]. Moreover, it is becoming apparent that there are clear phenotypic distinctions that can be drawn between tissue-resident cells and their non-resident counterparts [11, 12]. Therefore, there is a need for a deeper understanding of immune cell populations and their phenotypes in different tissues.

The lung presents a unique and challenging tissue context for immune populations. As a mucosal barrier, it is constantly exposed to environmental stimuli, necessitating robust defences against pollutants, particulates, and pathogens. However, in contrast to other mucosal surfaces and to facilitate gaseous exchange, this barrier, comprised of a single-cell layer, must remain in constant close contact with the innervating vasculature [13]. As a result, pulmonary immune responses are highly regulated, with dysregulated inflammation considered a hallmark of a wide variety of pulmonary disorders including asthma and chronic pulmonary obstructive disease (COPD) [14, 15]. Due to the challenges in obtaining airway samples, our understanding of the human pulmonary immune system is severely limited, in either health or disease.

Mononuclear phagocytes (MNPs)—including dendritic cells (DCs), macrophages, monocytes, and monocyte-derived cells—represent a highly heterogeneous and plastic population of cells with overlapping functions and surface marker expression [16, 17]. These MNPs can be further subdivided into an array of subsets based on cell surface expression of defining markers [16, 18]. MNPs are well represented in the lung, occupying both the alveolar and interstitial spaces as well the underlying parenchyma [13]. DCs in particular play a vital role in initiating and regulating immune responses to a wide variety of pathogens, environmental antigens, and self-antigens [16]. In humans, our understanding of MNPs is derived primarily from analysis of blood and skin samples [17, 19]. However, it is evident that there is substantial inter-tissue variation in the composition of the MNP compartment [20–22] meaning that these peripheral sites make for poor proxies of less accessible tissues. Whilst these cells have been implicated as major players in a variety of lung disease settings [23], our understanding of the composition of MNP populations in the human lung, its relationship to other tissues, and whether it is significantly modified in inflammatory disease settings is currently limited.

In partnership with MNPs, T cells function as major regulators of lung immune responses. CD4^+^ T cells are key sources of cytokines essential in feeding back to the myeloid compartment, coordinating adaptive immune responses, and supporting B-cell antibody responses [24]. CD8^+^ T cells are also capable of providing inflammatory cytokines and contribute to direct cytotoxic killing of infected cells [25], while CD4^+^FOXP3^+^ regulatory T cells (Tregs) are key regulators of inflammation in a wide variety of settings, essential for tissue protection [26, 27]. TCRγδ^+^ T cells also reside in the lung, but they remain poorly characterized and their function in lung tissue physiology is unclear even in murine systems [28]. In recent years, clear phenotypic differences between resident T cells, non-resident T cells, and peripheral blood T cells have been reported in a variety of tissue contexts, the delineation of which has centred around the expression of the canonical T cell tissue-residency markers CD69 and CD103 [11, 12, 29–33]. CD69, in particular, seems to demarcate long-lived tissue-resident memory (TRM) cells [24], with one elegant study showing that the majority of donor-derived CD4^+^ and CD8^+^ T cells from liver allografts express this marker for years following transplantation [31]. The requirement for CD103 in tissue retention is less clear as, whilst it is expressed on the majority of tissue-resident CD8^+^ T cells in the adult human intestines [30], its expression on CD4^+^ T cells and CD8^+^ T cells in other tissue compartments is more variable [33–35]. Additional complexity is presented by the fact that CD69 and CD103 are not always co-expressed within a given tissue, often with the concomitant presence of discrete CD69^+^CD103^−^, CD69^−^CD103^+^, and CD69^+^CD103^+^ T cell populations [34], suggesting that CD103 expression denotes T cell specialization correlated with—but independent from—tissue residence. Indeed, CD103^+^CD69^+^ CD8^+^ T cells in the liver have a phenotype distinct from CD103^−^CD69^+^ CD8^+^ T cells, expressing higher levels of chemokines, MHCII, and IL-2 [11].

At present, our understanding of composition and phenotype of the MNP and T cell compartments within the human lung is highly limited. This is particularly true in the context of chronic inflammatory disorders such as asthma and COPD. Few studies have performed detailed immune phenotyping on primary lung tissue and those that have are reliant on relatively small sample sizes [13]. More data is available for peripheral blood cells isolated from donors with such conditions, but it is unclear to what extent peripheral blood represents the pulmonary immune environment, meaning depth of understanding of the immune phenotype of lung-resident MNPs and T cells in these conditions is currently lacking.

To expand the scope of our understanding of the pulmonary immune environment, we performed flow cytometric phenotyping on cells isolated from resected lung tissue from 115 donors, with matched peripheral blood from 69 of these donors. We provide a detailed comparison of the immune phenotype of these two sites, identifying clear differences in the overall composition of immune populations and expression of phenotypic markers between lung tissue and blood sites. We demonstrate that pulmonary DCs express higher levels of activation markers compared to those in circulation and identify CD103 as a marker of recent receptor signalling in pulmonary CD4^+^ and CD8^+^ T cells. Finally, we demonstrate that, at resting state, the composition of the pulmonary MNP and T cell compartments is not significantly different in donors with asthma or COPD, relative to controls. Together, these data expand our overall understanding of the pulmonary immune network and its phenotypic distinction from peripheral blood, supporting a model in which immune dysregulation in the lung is a product of functional changes within cells rather than dramatic changes in the pulmonary immune cell landscape.

## Results

To interrogate the immune phenotype of human lung, we collected tissue resection samples from donors undergoing thoracic surgery and performed flow cytometric analysis on enzymatically digested lung single-cell suspensions from 115 donors (Table 1), 69 of whom we were also able to acquire PBMCs.

**Table 1:** Donor demographic data

Lung samples (*N* = 115)			Blood samples (*N* = 69)		
Gender	*N* (%)		Gender	*N* (%)	
Male	47 (41)		Male	29 (42)	
Female	68 (59)		Female	40 (58)	
Age	**Median (range)**		**Age**	**Median (range)**	
	71 (28–89)			72 (49–86)	
Disease group	***N* (%)**	**FEV1/FVC % mean (SD)**	**Disease group**	***N* (%)**	**FEV1/FVC % mean (SD)**
Control	60 (52.17)	77.0 (9.27)	Control	41 (59.42)	76.0 (6.08)
Asthma	17 (14.78)	81.8 (18.20)	Asthma	11 (15.94)	84.2 (21.60)
COPD	38 (33.04)	58.6 (10.60)	COPD	17 (24.64)	55.2 (12.20)
Smoking status	***N* (%)**	**FEV1/FVC % mean (SD)**	**Smoking status**	***N* (%)**	**FEV1/FVC % mean (SD)**
Never smoked	16 (13.91)	83.9 (17.80)	Never smoked	9 (13.04)	82.6 (15.1)
Ex-smoker	73 (62.61)	70.7 (13.10)	Ex-smoker	44 (63.77)	68.5 (13.1)
Current smoker	26 (22.61)	66.8 (13.10)	Current smoker	16 (23.19)	71.4 (15.3)
Disease group and smoking status	***N* (%)**	**FEV1/FVC % mean (SD)**	**Disease group and smoking status**	***N* (%)**	**FEV1/FVC % mean (SD)**
Control Never smoked	11 (9.57)	81.5 (17.40)	Control Never smoked	5 (7.25)	78.0 (4.74)
Control Ex-smoker	37 (32.17)	75.8 (5.64)	Control Ex-smoker	27 (39.13)	75.4 (5.65)
Control Current smoker	12 (10.43)	76.6 (7.40)	Control Current smoker	9 (13.04)	76.7 (8.10)
Asthma Never smoked	5 (4.35)	89.2 (19.40)	Asthma Never smoked	4 (5.80)	88.3 (22.30)
Asthma Ex-smoker	11 (9.57)	79.5 (18.30)	Asthma Ex-smoker	6 (8.70)	83.6 (24.30)
Asthma Current smoker	1 (0.87)	70.9	Asthma Current smoker	1 (1.45)	70.9
COPD Ex-smoker	25 (21.74)	59.2 (10.80)	COPD Ex-smoker	11 (15.94)	54.8 (13.70)
COPD Current smoker	13 (11.30)	57.5 (10.70)	COPD Current smoker	6 (8.70)	55.9 (9.99)

### Composition of the MNP compartment in the blood and lung

We first assessed the composition of the MNP compartment in the blood and lung by flow cytometry with a focus on DCs and monocytes. Plasmacytoid (p)DCs were identified as CD45^+^Lin^−^CD11c^-^MHCII^+^CD303^+^. The remaining DC and monocyte populations were identified as CD45^+^Lin^−^CD11c^+^MHCII^+^. Monocytes were then subdivided into either classical CD14^+^CD16^−^ monocytes, non-classical CD14^-^CD16^+^ monocytes, or CD14^+^CD16^int^ intermediate monocytes [36]. CD14^−^CD16^−^ cDCs were classified as CD1c^−^CD141^+^CD172a^−^ conventional (c)DC1s, CD1c^+^CD141^−^CD172a^+^CD1a^−^ cDC2s, or CD1c^+^CD141^−^CD172a^+^CD1a^+^CD206^+^ monocyte-derived (mo)DCs [37]. cDC2s were further subdivided based on CD206 expression (Supplementary Fig. S1).

**Figure 1:**
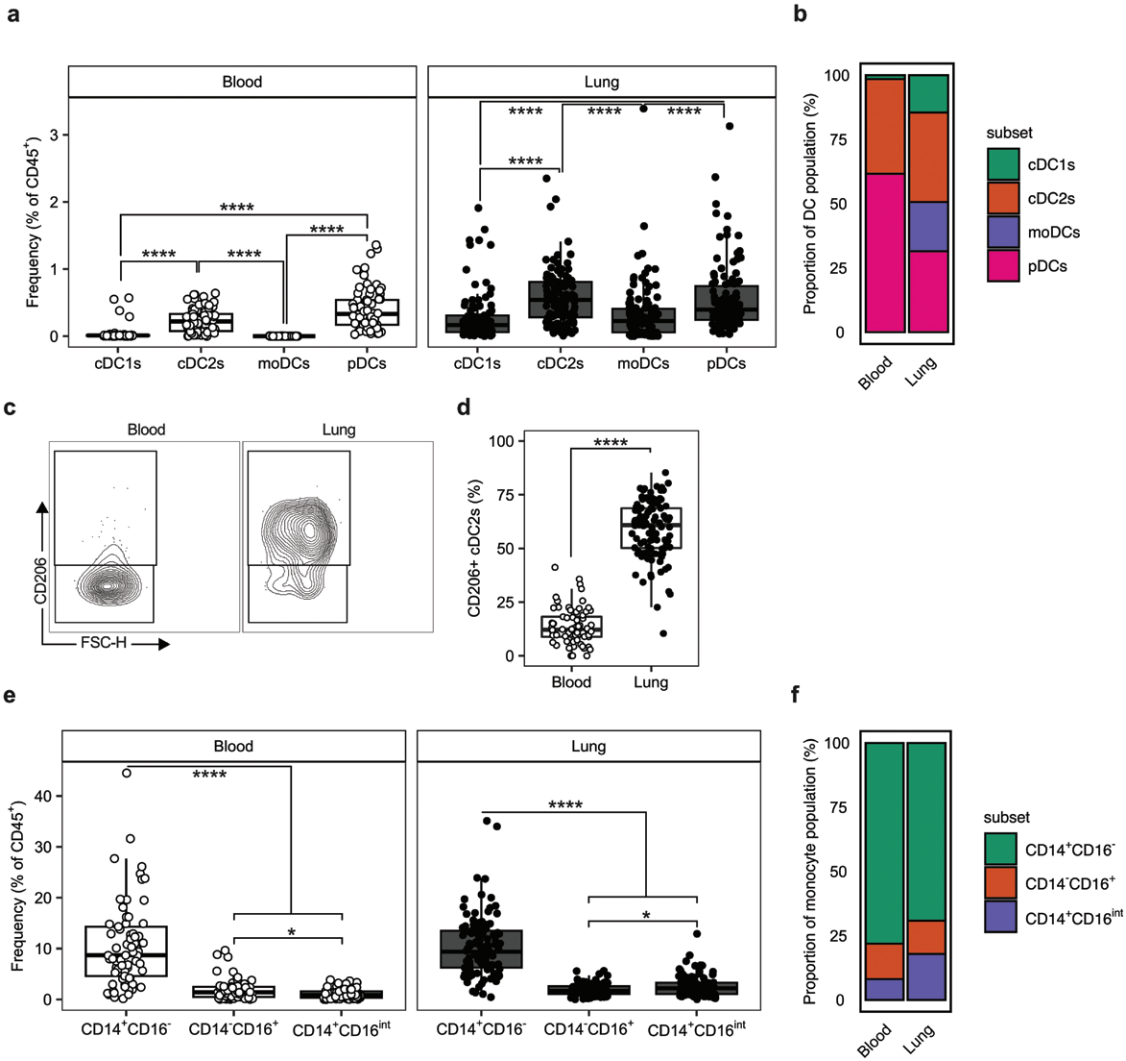
cDC2s are the dominant DC subset in the human lung. (**a)** Quantification of DC subsets in the human lung (*n* = 114) and peripheral blood (*n* = 69) given as the frequency of CD45^+^ cells. (**b)** Stacked bar plots representing DC subsets as a proportion of the total DC population in the blood and lung. (**c)** Representative contour plots showing the expression of CD206 on cDC2s in the blood and lung with (**d)** quantification given as percentage (%) of total cDC2s expressing CD206. (**e)** Quantification of monocyte subsets in the human lung and peripheral blood given as the frequency of CD45^+^ cells. (**f)** Stacked bar plots representing monocytes subsets as a proportion of the total monocyte population in the blood and lung. Box plots indicate median and interquartile range, whiskers represented the maximum/minimum value within 1.5× the upper/lower quartile limit. Dots indicate the values of individual donors. *P < 0.05; **** *P* < 0.0001. *P* values were calculated by unpaired Wilcoxon two-sample tests and adjusted for multiple comparisons using the Holm method.

We observed cDC2s and pDCs to be the dominant DC populations in lung, existing at significantly greater frequency than cDC1s and moDCs (Fig. 1a, Supplementary Fig. S2a). Although this pattern was consistent in the blood pDCs, were present at significantly greater frequency than cDC2s in this compartment (Fig. 1a, Supplementary Fig. S2a). Consistent with previous literature [19], cDC1s represented a small fraction of peripheral blood DCs and accounted for less than 1% of total PBMCs (Fig. 1b). The C-type lectin receptor CD206 (mannose receptor) is a pattern recognition receptor (PRR) associated with signalling by type-2 cytokines, particularly IL-4 and IL-13 [38], and used as a moDC marker on CD1a+ cells [37]. We noted that CD206 expression on cDC2s was dramatically increased in the lung relative to the blood (Fig. 1c and d), indicating expression is dependent on cells that are associated with the lung environment.

Analysis of monocytes showed that CD14^+^CD16^−^ (classical) monocytes were the dominant subset (vs. CD14^−^CD16^+^ and CD14^+^CD16^int^ populations) in both lung and blood (Fig. 1e and f). Of these smaller subsets, we observed a small but statistically significant difference between the frequency of CD14^+^CD16^−^ and CD14^+^CD16^Int^ monocytes present in both the blood and lung (Fig. 1e, Supplementary Fig. S2b). In the blood, CD14^−^CD16^+^ monocytes were present at higher frequency whereas the reverse was true in the lung (Fig. 1f).

### The lung environment promotes the expression of activation markers by myeloid cells

**Figure 2:**
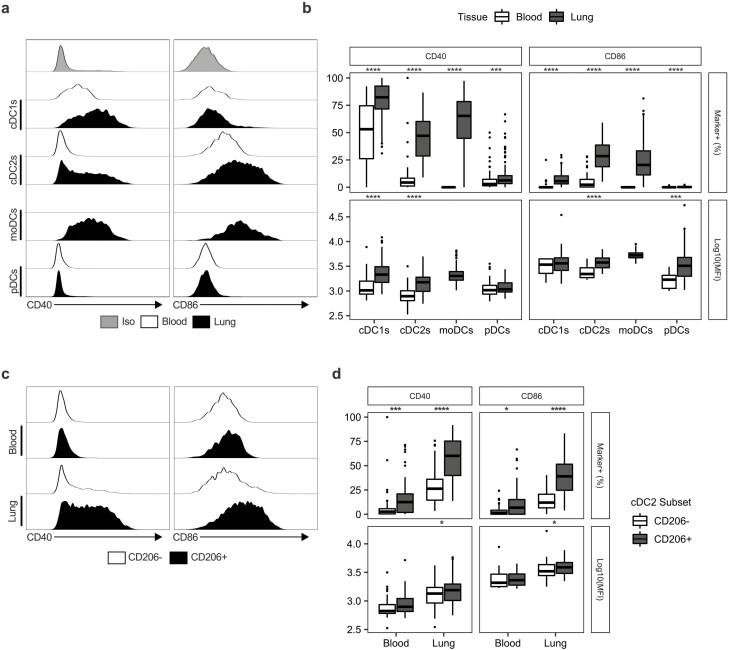
Expression of maturation markers on DCs is increased in the lung relative to the blood. (a) Representative histograms showing the expression of CD40/CD86 on DC subsets in the lung (*n* = 114) and blood (*n* = 69). Iso indicates matched isotype negative control staining on pooled lung cells. (**b)** Quantification of CD40/CD86 expression on DC subsets given as the frequency of cells positive for each marker and the Log10 of the median fluorescent intensity (MFI). (**c)** Representative histograms of CD40/CD86 expression on CD206^+^ and CD206^-^ cDC2s in the blood and lung. (**d)** Quantification of CD40/CD86 on CD206^+^ and CD206^−^ cDC2s expressed as the frequency of cells positive for each marker and the Log10 of the median fluorescent intensity (MFI). Box plots indicate median and interquartile range, whiskers represented the maximum/minimum value within 1.5× the upper/lower quartile limit. Points beyond the whiskers indicate outliers. *P* values were calculated by unpaired Wilcoxon two-sample test and adjusted for multiple comparisons using the Holm method. *P < 0.05; ***P < 0.001; ****P < 0.0001.

To assess whether pulmonary myeloid cells in the lung displayed a different level of activation compared to their counterparts in circulation, we measured expression of the canonical myeloid maturation markers CD40 and CD86 as well as MHCII (Fig. 2, Supplementary Fig. S3).

For all DC subsets captured by our panel, we observed significant increases in the frequency of expression of both CD40 and CD86 in lung DCs when compared to blood, which was particularly pronounced in cDC2s (Fig. 2a and b). In cells positive for these markers, we detected significant increases in the median fluorescence intensity (MFI) of CD40 on cDC1s and cDC2s, and of CD86 on cDC2s and pDCs, in the lung relative to the blood (Fig. 2b). Conversely, when assessing the expression of MHCII we found that DCs isolated from the lung tissue expressed significantly lower levels than those isolated from the blood (Supplementary Fig. S3b). Strikingly, when we compared CD206^+^ cDC2s with those that did not express CD206 we observed a significant increase in the frequency of CD40 and CD86 expression by CD206^+^ cells (Fig. 2c and d). This increase in CD40/CD86 positivity was noticeable in the blood (Fig. 2d) but was substantially greater in the lung, indicating that CD206 expression may demarcate a mature population of cDC2s.

Changes in expression of activation markers were less pronounced in monocytes (Supplementary Fig. 3a). However, we observed statistically significant increases in the frequency of CD40 expressing CD14^+^CD16^−^ and CD14^+^CD16^int^ monocytes, as well as significant increases in the MFI of CD40 in all monocyte populations (Supplementary Fig. S3a). In contrast to DCs, we detected no change in the frequency of monocytes expressing CD86 in the lung relative to the blood, although there was a small but statistically significant increase in the MFI of CD86 on CD14^+^CD16^int^ monocytes in the lung relative to the blood. Additionally, CD14^+^CD16^−^ monocytes expressed higher levels of MHCII in the lung tissue relative to peripheral blood, whilst CD14^-^CD16^+^ monocytes had reduced MHCII expression (Supplementary Fig. S3c).

### Differential expression of residency markers is associated with distinct T-cell phenotypes

**Figure 3:**
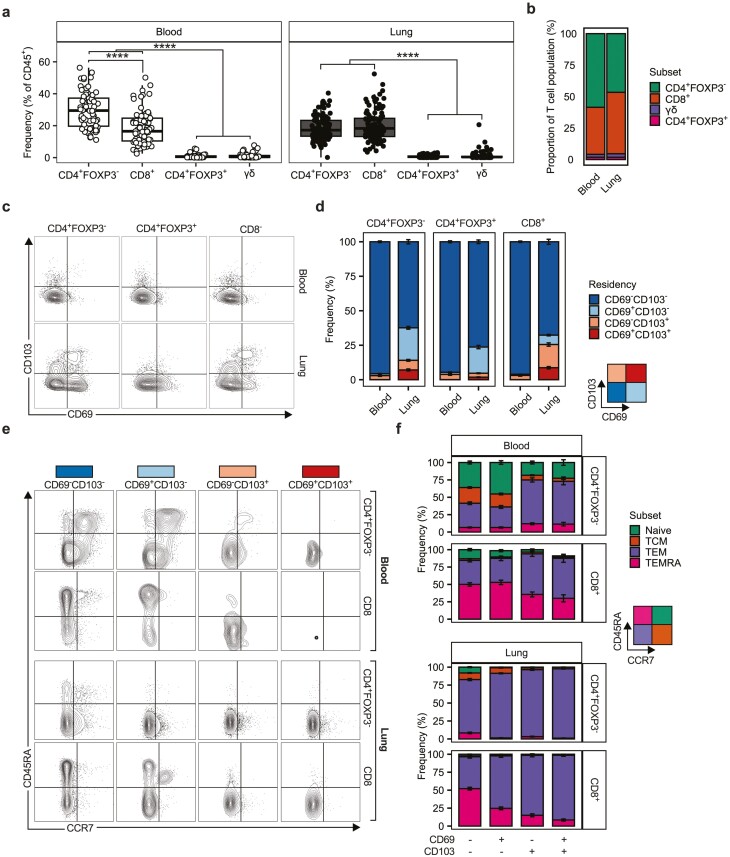
Tissue compartment and expression of residency markers define T cell phenotype. (**a)** Quantification of T cell (SCC-A^low^CD45^+^CD3^+^ cells) subsets in the human lung (*n* = 112) and peripheral blood (*n* = 68) given as the frequency of CD45^+^ cells. γδ T cells were identified as TCRγδ^+^, CD4^+^FOXP3^−^ T cells were identified as TCRγδ^-^CD4^+^CD8^−^FOXP3^−^, CD4^+^FOXP3^+^ T cells were identified as TCRγδ^−^CD4^+^CD8^−^FOXP3^+^, and CD8 T cells were identified as TCRγδ^−^CD4^−^CD8^+^. Points represent individual donors. Box plots indicate median and interquartile range, whiskers represent the maximum/minimum value within 1.5× the upper/lower quartile limit. (**b)** Stacked bar plots representing the T cell subsets as a proportion of the total T cell population in the lung and blood. (**c)** Representative contour plots showing expression of putative residency markers (CD69 & CD103) in T cell subsets in the lung and blood with (**d)** quantification represented as stacked bar graphs. (**e)** Representative contour plots of CD45RA/CCR7 expression on blood and lung CD4^+^FOXP3^−^ and CD8^+^ T cells residency marker subsets. CD45RA/CCR7 expression defined four T cell phenotypes: Naive (CD45RA^+^CCR7^+^), T-central memory (TCM, CD45RA^−^CCR7^+^), T-effector memory (TEM, CD45RA^−^CCR7^−^), and T-effector re-expressing CD45RA (TEMRA, CD45RA^+^CCR7^−^). (**f)** Quantification of the frequency of CD4^+^FOXP3^−^ and CD8^+^ T cell phenotypes in residency marker subsets in the blood and lung. Representative contour plots are from five concatenated control donors. Stacked bars represent the mean frequency of a given population across the entire dataset. Error bars indicate the standard error of the mean. *P* values were calculated by unpaired Wilcoxon two-sample test and adjusted for multiple comparisons using the Holm method. ****P < 0.0001.

In tandem with and coordinated by DCs, T cells represent major regulators of immune responses. Given that DCs presented a distinct profile in the lung compared to the blood (Figs. 1 and 2), we undertook phenotyping of the T cell compartment (Supplementary Fig. S4) in these sites to identify whether comparable distinction in T cell phenotype could be observed between locations. CD4^+^FOXP3^−^ and CD8^+^ T cells were the dominant T cell populations in the lung (Fig. 3a), present at comparable frequencies (Supplementary Fig. S5a). As expected, CD4^+^FOXP3^+^ and γδ T cells were found with substantially less frequency than CD4^+^FOXP3^-^ or CD8^+^ T cells, but at comparable levels to one another (Fig. 3a, Supplementary Fig. S5a). This pattern was similar in the blood (Fig. 3a) although CD4^+^FOXP3^−^ cells were present at a statistically greater frequency than CD8^+^ cells at this site (Fig. 3b, Supplementary Fig. S5a).

CD69 and CD103 have been used to delineate tissue residency for T cells in a number of sites [34], with CD69 expressing CD4^+^ T cells within tissues thought to represent a distinct subset phenotypically distinct from circulating T cells [29]. We observed increased CD69 and CD103 expression in the lung relative to the blood for CD4^+^FOXP3^−^, CD4^+^FOXP3^+^, and CD8^+^ T cells, although CD69^−^CD103^−^ cells still accounted for >50% of all T cells present (Fig. 3c and d, Supplementary Fig. S5b). Notably, whilst CD69 or CD103 expression was low in the blood, a clear population of CD69^−^CD103^+^ cells was observed in this site, whilst CD69^+^CD103^+^ and CD69^+^CD103^−^ cells were virtually undetectable (Fig. 3d). This observation was consistent for CD4^+^FOXP3^−^, CD4^+^FOXP3^+^, and CD8^+^ T cells (Fig. 3d).

As expression of CD69 and/or CD103 has been associated with an effector memory phenotype [34, 39], we analyzed CD4^+^FOXP3^−^ and CD8^+^ T cells based on expression of CD69 and CD103, dividing them into four groups (CD69^−^CD103^−^, CD69^+^CD103^−^, CD69^−^CD103^+^, CD69^+^CD103^+^), and assessed their expression of CD45RA and CCR7. Differential expression of CD45RA and CCR7 yielded four T cell phenotypes: naïve T cells (CD45RA^+^CCR7^+^), central memory T cells (TCM, CD45RA^−^CCR7^+^), effector memory T cells (TEM, CD45RA^−^CCR7^−^), and effectors re-expressing CD45RA T cells (TEMRA, CD45RA^+^CCR7^−^) [34] (Fig. 3e). We observed significant differences in the frequencies of different CD4^+^FOXP3^−^ and CD8^+^ T cell phenotypes based on expression of CD69 or CD103 both in the blood and the lung (Fig. 3f, Supplementary Fig. S5c). Notably CD103 expression, on its own or in conjunction with CD69, was associated with an increase in the frequency of CD4^+^FOXP3^−^ and CD8^+^ T cells with a TEM phenotype, and a concomitant decrease in those with a naïve phenotype, in both sites (Fig. 3f).

### CD103 defines a TCR and IL-7R signalling experienced population of effector memory T cells in the lung

Given that differential expression of CD69 and CD103 was associated with differing phenotypes in both CD4^+^FOXP3^−^ and CD8^+^ T cells, of which TEMs were dominant in the lung, we assessed whether the expression of these markers was associated with other readouts of phenotypic activation in CD4^+^FOXP3^−^ and CD8^+^ TEMs. To do this we measured expression of the TCR co-stimulatory receptor, CD28, and the IL-7R, CD127. Since downregulation of these receptors is associated with experience of TCR and IL-7R signalling respectively, cells expressing these receptors are likely to be unstimulated, while decreased expression can be associated with recent activation [12, 34].

**Figure 4:**
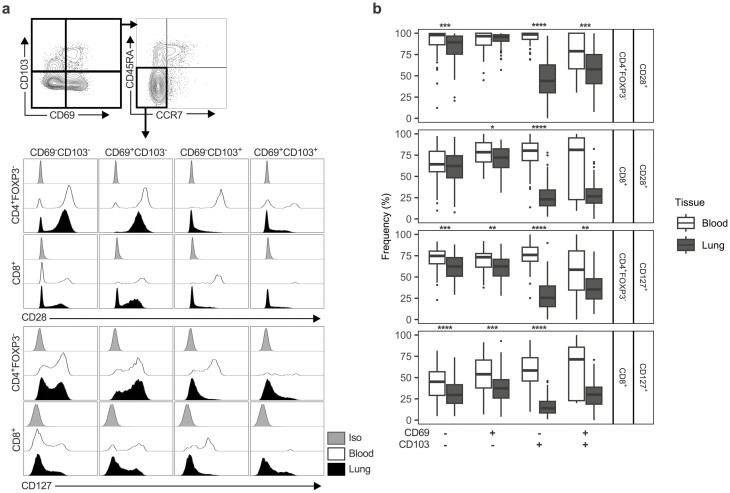
CD103 demarcates a TCR and IL-7R signalling experienced subset of CD4 and CD8 TEM cells in the human lung but not in peripheral blood. (**a**) Representative gating strategy for identification of CD4^+^FOXP3^−^ and CD8^+^ TEM cells (CD45RA^−-^CCR7^−^) from residency marker subsets with representative histograms showing CD28 and CD127 expression on these subsets in the lung (*n* = 112) and the blood (*n* = 68). Iso indicates matched isotype negative control staining on pooled lung cells. Plots are derived from five concatenated control donors. (**b)** Quantification of CD28/CD127 expression on CD4^+^FOXP3^−^ and CD8^+^ TEM residency marker subsets given as percentage of cells expressing each marker. Box plots indicate median and interquartile range, whiskers represented the maximum/minimum value within 1.5× the upper/lower quartile limit. Points beyond the whiskers indicate statistical outliers. *P* values were calculated by unpaired Wilcoxon two-sample test and adjusted for multiple comparisons using the Holm method. *P < 0.05; **P < 0.01; ***P < 0.001; ****P < 0.0001.

We observed significant differences in expression of both CD28 and CD127 on CD4^+^FOXP3^-^ and CD8^+^ TEMs between blood and lung, with the lung generally demonstrating lower expression of these markers (Fig. 4a and b), suggesting increased T cell activation within the lung tissue. Interestingly, CD103 expressing (either CD69^−^CD103^+^ or CD69^+^CD103^+^) CD4^+^FOXP3^−^ and CD8^+^ TEM cells showed the lowest levels of CD28 and CD127 expression relative to the blood, and compared to other lung TEMs not expressing CD103 (Fig. 4b, Supplementary Fig. S6). These data indicate that, whilst CD103 expressing TEMs formed a smaller fraction than CD103^-^ TEMs, they represented the portion of TEMs with TCR and/or IL-7R signalling experience.

### Chronic pulmonary inflammatory disorders are not associated with significant changes in cellular immune composition of the lung tissue

**Figure 5:**
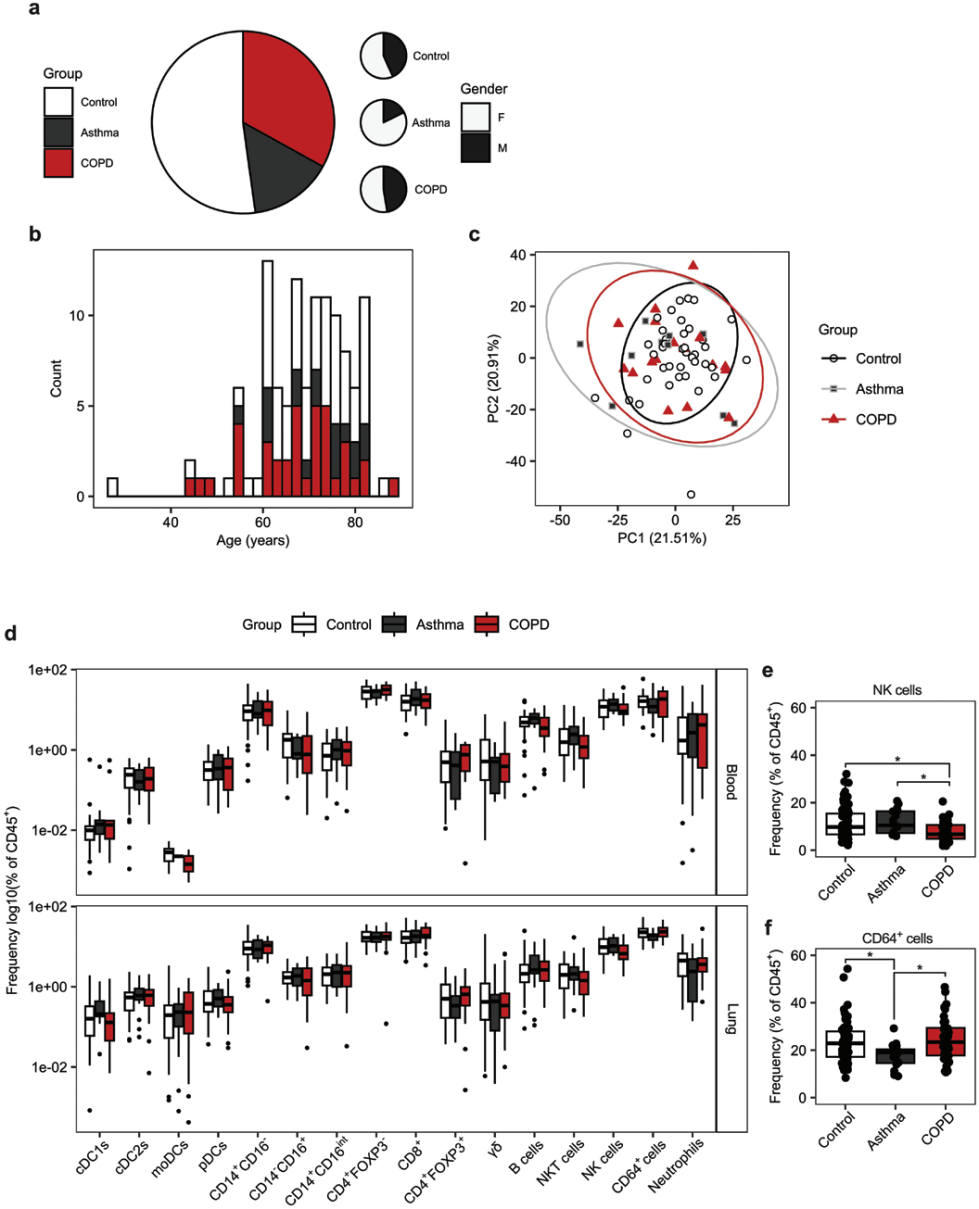
Asthma and COPD are not associated with gross changes to the lung immune compartment. (**a**) Pie charts representing the proportion of donors with asthma donors: *n* = 17, median age = 72 year, age range = 55–83 year, 14 female or COPD (donors: *n* = 38, median age = 69 year, age range = 45–83 year, 23 female) and the gender ratio within groups. Donors were grouped as control if they were not identified to have asthma, COPD, or a history of other inflammatory disorders (donors: *n* = 60, median age = 71, age range = 28–86, 34 female). (**b)** Histogram representing the age distributions for control, asthmatic, and COPD donors. (**c)** Principle components analysis (PCA) plot of flow cytometry data. Points represent individual donors, shape and colour denote group. (**d)** Boxplots representing the frequency of immune cell subsets in peripheral blood and lung tissue separated by group. Quantification is given as the Log10 of the frequency of CD45^+^ cells. (**e and f)** Boxplots representing the frequency of (e) NK cells and (f) CD64^+^ cells in the lung tissue given as the frequency of CD45^+^ cells. Boxplots indicate the median and interquartile range, whiskers indicate the maximum/minimum value no greater/lesser than 1.5× the quartile limit. Points beyond the whiskers indicate statistical outliers. *P* values were calculated by unpaired Wilcoxon two-sample test and adjusted for multiple comparisons using the Holm method. *P < 0.05.

As a major immune barrier site, immunological dysregulation within the lung is associated with a variety of pulmonary disorders including asthma, COPD, and pulmonary fibrosis [14, 15, 40]. Within our cohort, two of the most prevalent conditions observed were asthma and COPD, accounting for 14.78% (*n* = 17) and 33.04% (*n* = 38) of subjects, respectively (Fig. 5a). Donors not suffering asthma or COPD, and who had not previously been excluded due to a history of other inflammatory disease, were treated as controls. Both men and women were represented in each group, although all groups had a greater proportion of women, with this being particularly pronounced in the asthma group (Fig. 5a). The distribution of ages between groups was not significantly different, as determined by Kolmogorov–Smirnov test (control vs. asthma *P* = 0.9906, control vs. COPD *P* = 0.6789, asthma vs. COPD *P* = 0.4049) (Fig. 5b). Comparable numbers of cells were isolated from donor lung tissue regardless of disease group or smoking status (Supplementary Fig. S7a). The majority of donors in each group were within the clinically typical blood neutrophil (1.8–7.5 × 10^9^ cells/l) and blood eosinophil (0.0–0.4 × 10^9^ cells/l) ranges (Supplementary Fig. 7a) and statistical comparison did not identify significant differences in the frequency of immune cell populations between donors that exceeded the typical range and those within it for both neutrophils and eosinophils.

To determine whether asthma or COPD had a significant effect on the frequency of blood and lung immune cells we compared a range of immune cell types between groups. We did not observe a statistical difference in the frequency any of the DC, monocyte, or T cells subsets assessed previously, nor in the total number of B cells (SSC-A^low^CD45^+^CD3^−^CD19^+^), Neutrophils (SSC-A^high^CD45^+^CD64^−^CD66b^+^CD11b^+^CD16^+^), or NKT (SSC-A^low^CD45^+^CD19^−^CD3^+^CD56^+^) cells when comparing asthmatic and COPD donors to controls in both peripheral blood and lung tissue (Fig. 5d). However, we identified a significant reduction in the frequency of NK cells (SSC-A^low^CD45^+^CD19^−^CD3^−^CD56^+^) in the lung tissue of COPD donors relative to control and asthmatic donors (Fig. 5e). Additionally, there was a small but significant reduction in the frequency of CD64^+^ cells (SSC-A^int^CD45^+^CD66b^−^CD64^+^) in the lung tissue of asthmatic donors relative to control and COPD donors (Fig. 5f). However, despite these differences, when overall composition was assessed by principle components analysis, we did not observe donor samples clustering by group, and multivariate statistical comparison by permutational ANOVA indicated that group did not have a significant effect on overall cellular immune composition (*P* = 0.715) (Fig. 5c).

We next assessed whether we could detect phenotypic differences in the MNP and T cell compartments between groups. However, consistent with cell subset frequencies, we did not observe any significant differences in expression of CD40, CD86, or MHCII on MNP subsets between groups (Supplementary Fig. S7c–f). Similarly, the expression of residency markers and of CD28 or CD127 on T cell subsets was not statistically different between groups (Supplementary Fig. S8). Collectively, these data indicate that asthma and COPD were not associated with substantial changes to the gross composition of the lung immune cell compartment.

Innate lymphoid cells (ILCs) have recently been recognized as major players in the regulation of mucosal barrier responses, largely through work in murine models [41, 42]. Little is known as to how ILCs are affected by asthma or COPD. Although previous studies have associated COPD with an increased frequency of ILC1s in the lungs and peripheral blood of donors relative to non-smoking controls [43, 44], earlier data showed no difference in ILC1s and instead indicated that group 3 ILCs, which do not express natural cytotoxicity receptor (NCR^-^ILC3s) are increased in the lungs of COPD donors [45]. To increase our fundamental understanding of ILCs in pulmonary disorders, we assessed the frequency of ILCs (Lin^-^CD56^−^CD127^+^ cells) in a subset of our cohort (96 donors) by flow cytometry (Supplementary Fig. S9a). We found ILC1s to be the most common ILC subset present in the human lung, accounting for 42% (mean, SEM = ±2.1%) of total ILCs (Supplementary Fig. S9b). However, in keeping with the other cell types we assessed (Fig. 5), we observed no statistical difference in the total number of pulmonary ILCs between controls and asthmatic or COPD donors (Supplementary Fig. S9c), nor did we identify a statistical difference in the frequency of any ILC subset between these groups (Supplementary Fig. S9d). In contrast to the lack of impact of asthma or COPD on lung cellularity, when we subdivided our donors based on their smoking status we observed a significant increase in the frequency of NCR^+^ILC3s in control and COPD donors who self-identified as current smokers when compared to never smoker controls (Supplementary Fig. S9e). We also observed a non-significant trend for a reduction in the frequency of ILC2s in current smoker COPD donors (Supplementary Fig. S9e), consistent with previous results in peripheral blood [44]. Contrary to previous reports [43, 44] we did not detect a robust difference in the frequency of ILC1s in COPD donors and smoking controls compared to non-smoking controls in our cohort (Supplementary Fig. S9d).

Given the effect of being a current smoker on NCR^+^ILC3s in COPD donors, we asked whether smoking had any effect on the frequency of other cell types in the lung. As we only identified one current smoker asthma donor and no never smoker COPD donors in our cohort, these groups were not included in our analysis. PCA analysis did not identify clustering based on smoking status (Supplementary Fig. S10a). However, we observed significant reductions in the frequency of cDC1s present in the lungs of current smokers in both control and COPD donors compared to non- or ex-smokers in each group, respectively. Additionally, in COPD donors we observed reduced frequencies of CD14^−^CD16^+^ monocytes, CD14^−^CD16^int^ monocytes, and CD4^+^FOXP3^−^ T cells in the lungs, but not the blood, of current smokers compared to ex-smokers. These data indicate that the effects of smoking on lung immune cell populations are limited to certain specific subsets and are not readily identifiable by profiling of PBMCs.

## Discussion

Our understanding of the immune compartment in the human lung remains limited in scope due to the relative inaccessibility of tissue samples in healthy donors and many disease states. Although studies on cellular responses in donors during immune activation tend to be restricted to peripheral blood, the extent to which the blood reflects the immune phenotype of the lung is also poorly understood. Thus, it is challenging to parse which observations in the periphery can be extrapolated to the lung itself and which cannot. To directly address this important and understudied area, we performed flow cytometric immune phenotyping on single-cell suspensions from lung tissue resections and matched peripheral blood. We identified significant differences in the expression of maturation and residency markers in major myeloid and T cell subsets between the peripheral blood and lung tissue compartments. We observed a strong upregulation of the activation markers CD40 and CD86 on lung DCs compared to those in circulation and identified CD206 as a marker of maturation in lung cDC2s. Within the T cell compartment, we linked CD103 expression in lung CD4 and CD8 TEMs with experience of IL-7R and TCR signalling. Finally, we demonstrated that the general composition of the pulmonary T cell, monocyte, DC, and ILC compartments of asthmatic and COPD donors are comparable to controls.

MNPs are a highly heterogeneous population with overlapping functions and expression of cell surface markers, making accurate delineation of populations by conventional flow cytometry challenging [17, 46], further complicated by tissue-specific expression of markers in different myeloid subpopulations [16, 17, 36]. To gain an overview of the MNP compartment in the human lung, we applied a conservative gating strategy utilizing accepted markers of monocyte and DC subpopulations [21, 22] to distinguish four major DC subsets and three monocyte subsets. Consistent with recent observations using single-cell sequencing [47], we identified cDC2s as the primary DC population in the lung, closely followed by pDCs, although both cDC1s and moDCs were also represented in this compartment. We detected a strong increase in the expression of CD40 and CD86 on all DC subsets in the lung compared to those in circulation, suggesting that the lung environment facilitates DC maturation either as a consequence or in anticipation of exposure to environmental antigens.

We also identified differential expression of CD206 on cDC2s between the lung and the blood, with the vast majority of lung cDC2s expressing CD206 whilst the majority of blood DCs did not express this marker. Interestingly, CD206^+^ cDC2s in the lung expressed significantly higher levels of CD40 and CD86 than their CD206^−^ counterparts (Fig. 2). A previous report, that elegantly distinguished between pulmonary monocytes and DCs within the lung epithelium/stroma and those in the pulmonary vasculature, demonstrated that CD206 expression on pulmonary monocytes and DCs is restricted to extravascular cells [48]. This suggests that, in our dataset, CD206^−^ lung cDC2s may represent cDC2s within the pulmonary vasculature and supports CD206 as a potential marker of tissue residency in cDC2s. CD206, also known as the mannose receptor, recognizes a broad array of organisms spanning fungi, bacteria, and protists [49], with its specific expression on tissue-resident DCs implying a necessity for this receptor in the recognition of and response to invading organisms. Early investigation into expression of CD206 using *in vitro* derived DCs correlated LPS-induced maturation with down-regulation of CD206 [50]. We demonstrate that, relative to immature circulating cDC2s, CD206 is linked to increased frequency of CD40 and CD86 expression by cDC2s in the lung tissue. However, we cannot rule out that following stimulation CD206 expression is lost on DCs as they egress the lung. Irrespective, our data support clear phenotypic differences between circulating and tissue-resident DCs and elevate basic understanding of the lung myeloid compartment in this regard.

In terms of T cells, we identified pulmonary CD103^+^CD4^+^FOXP3^−^ and CD103^+^CD8^+^ TEMs as being those with recent experience of IL-7R and TCR signalling, as determined by substantial downregulation of CD28 and CD127 expression within these T cell subpopulations (Fig. 4). This suggests that expression of CD103 is linked to recent T cell activation within the lung. Consistent with our results, a recent report demonstrated that pulmonary CD103^+^CD4^+^ cells have a distinct phenotype from CD103^−^CD4^+^ T cells, expressing a different pattern of chemokine receptors and exhibiting a greater capacity for IFNγ and TNFα production following stimulation [51]. Similarly, a previous analysis of CD103 expression on CD4^+^ TRM cells present in female reproductive tissue found that CD103^+^ cells had a greater capacity to produce IL-17A than CD103^-^CD4^+^ cells following *ex vivo* PMA/ionomycin stimulation. This observation did not extend to canonically non-Th17-associated cytokines such as IFNγ, suggesting that CD103 denotes a Th17 signature at this barrier site and a specialized need for regulation of the local bacterial microbiota [32]. In agreement with this observation, CD103^+^CD69^+^ liver CD8^+^ T cells have been shown to produce greater levels of IL-2 than their CD103^-^ counterparts whilst expressing comparable levels of IFNγ [11]. Similarly, CD69^+^ CD8^+^ T cells in the brain can be divided into CD103^+^ and CD103^−^ populations with distinct phenotypes [12]. In an elegant study of long-lived T cell populations in the liver following transplantation, it was observed that, whilst the majority of donor-derived CD4^+^ T cells express CD69, they display minimal CD103 expression [31], suggesting that CD103 is not required for long-term tissue retention or residency status of CD4^+^ T cells. However, a study of T cells isolated from the bronchioalveolar lavage of HLA mismatched lung transplant recipients demonstrated that 9 months post-transplantation the majority of donor T cells remaining in the airways were CD69^+^, with a substantial proportion of those cells also expressing CD103 [52]. These populations existed at significantly greater frequency than at 1-month post-transplantation, indicating a correlation between airway retention and CD103 expression over time, although the presence of a significant proportion of CD69^+^CD103^−^CD4^+^ T cells would again suggest that CD103 is not required for tissue retention [52]. Interestingly, we observed comparable levels of CD28 and CD127 expression on pulmonary CD69^-^CD103^+^ and CD69^+^CD103^+^ CD4^+^FOXP3^−^ and CD8^+^ TEM cells (Fig. 4), perhaps implying that these lung CD69^−^ cells represent non-resident, recently recruited, TEMs following TCR stimulation. Alternatively, these CD69^−^CD103^+^ CD4^+^FOXP3^−^ T cells may represent resident cells down-regulating their residency program to facilitate egress from the tissue environment. Further, we observed small CD103^+^CD4^+^FOXP3^−^ and CD103^+^CD8^+^ T cell populations in peripheral blood, the majority of which had a TEM phenotype (Fig 3). However, unlike pulmonary T cells, these cells expressed high levels of CD28, implying a lack of recent TCR stimulation (Fig 4). A previous RNAseq analysis found that CD103^+^ blood CD8^+^ T cells were transcriptionally more similar to CD103^−^ blood CD8^+^ T cells than CD103^+^ pulmonary CD8^+^ T cells, suggesting that these cells do not represent CD8^+^ TRM cells which have recently egressed from the lung [53]. However, whether CD103^+^ blood T cells represent a population in-transit to various tissue environments or a distinct circulating population remains unclear. Collectively, these data support a model in which, independent of CD69 expression, CD103 expression on lung TEM cells marks a subpopulation of active tissue specialists with recent receptor signalling experience and functional adaptation to the tissue environment in which they are located.

Both asthma and COPD are inflammatory disorders of the airways associated with immune dysfunction [14, 15]. Allergic asthma, the primary asthma endotype, is driven by an inappropriate and excessive eosinophilic Th2 response to harmless environmental stimuli (Th2-high asthma) [54]. Those donors with non-allergic/non-eosinophilic asthma (Th2-low asthma) present with greater neutrophilia and a resistance to steroid treatment [55]. Similarly, as an inflammatory condition COPD is triggered by external factors, commonly viral and bacterial infection, which drive CD8^+^ T cell and neutrophilic inflammation [55]. However, there is currently little understanding as to what extent these conditions reshape the lung immune compartment. We identified 17 donors with asthma and 38 with COPD within our cohort through a combination of self-reporting and clinical presentation. Somewhat unexpectedly, we did not observe major differences in the number of cells per gram of tissue when assessing a wide range of cell types and subsets in asthmatic and COPD donors relative to controls, although we noted a small reduction in NK cells in COPD donors and reduced frequency of CD64^+^ cells in asthmatics (Fig. 5). Further, we could not detect differences in the expression of the maturation markers CD40 and CD86 in myeloid population, nor differences in the expression of residency (CD69 or CD103) or phenotypic markers (CD45RA, CCR7, CD28, CD127) on T cell subsets (Supplementary Figs. 7 and 8).

A previous study observed statistical increases in the frequency of CD4^+^ and CD8^+^ T cells in the lungs of COPD donors as a proportion of the CD45^+^ compartment, as well as an increase in moDCs as a percentage of their CD11b^+^CD11c^+^HLA-DR^+^ DC fraction [56]. Notably, the authors identified accumulation of T and B lymphocytes in regions of airways of COPD donors where the secretory immunoglobulin A (SIgA) had been lost [56], suggesting that major changes in the immune landscape of the lung occur in highly localized fashion which may not be reflected in bulk analysis by flow cytometry. It has also been observed that cDC2s isolated from COPD donors have a distinct transcriptional profile, an increased capacity to induced naïve CD4^+^ T cell differentiation into a T follicular helper phenotype, and localize to distinct tissue regions relative to non-COPD controls [46]. An analysis of blood and bronchiolar lavage reported a reduced frequency of γδ T cells in COPD donors, although this observation was only evident when comparing COPD donors with smokers not presenting with COPD, as COPD donors showed statistically comparable levels of γδ T cells to non-smoker controls [57]. Notably, however, these studies used relatively small numbers of donors and/or focused on small, localized differences. The data presented here takes a broader view, capturing the overall lung phenotype in over 100 donors, which may explain differences in our observations.

A role for ILCs in COPD, and more generally in response to cigarette smoke and environmental pollutants, is emerging [58]. However, there is little consensus on how the ILC compartment is affected by COPD. The evidence that is available has indicated that either ILC1s or NCR^-^ILC3s are increased in the lungs of donors with COPD [43, 45]. However, these studies have relied on relatively small sample sizes, especially with regard to the non-COPD controls. In the 96 donors within our cohort profiled for ILC phenotype, we could not observe statistically significant differences in the frequencies of ILC1s, NCR^−^ILC3s, or ILC2s in COPD donors relative to non-COPD controls. Indeed, differences only became apparent when donors were separated based on smoking status, where we observed a trend to a reduction in ILC2 frequency in COPD donors relative to non-smoking controls and an increase in the frequency of NCR^+^ILC3s in ex and current smokers, especially those with COPD. These inconsistencies between studies are likely a result of high inter-person variability, small sample sizes, and differences in flow cytometric gating strategies used to identify ILC subsets. Further compounding the difficulty in linking changes in the ILC compartment with COPD is the recent observation that NCR^+^ILC3s accumulate in the lungs of non-small cell carcinoma donors [59]. Therefore, as the majority of primary lung tissue—including that used in this study—is only accessible from resected tissue proximal to tumour sites, disentangling whether NCR^+^ILC3 recruitment to the lung, or indeed any changes in ILC subset frequency, is a consequence of smoking, COPD, or the proximity to a tumour site is challenging.

Our data from the lungs of asthmatic donors are consistent with previous analyses of MNPs in the blood [60] and sputum [61] which reported either no difference or only weakly significant minor changes in the frequencies of various MNP subsets at steady state, although more robust differences could be observed following allergic challenge [61, 62]. It has been reported that DCs expressing BDCA1 can be found specifically within the airway epithelium of asthmatics with a Th2-high phenotype, but not in those with a Th2-low phenotype [63]. The highly localized nature of this observation, and its specificity to Th2-high asthma, implies that remodelling of the immune compartment at a steady state in asthmatics is likely localized to specific tissue structures and is dependent on the endotype of a given donor. Consistent with our data, a recent single-cell analysis of human lung biopsies from asthmatics did not identify major changes in the overall composition of the immune compartment in terms of frequency of major immune cell lineages relative to non-asthmatic controls [64]. However, the authors identified significant changes to the cell–cell signalling landscape of the asthmatic lung at a transcriptional level, supporting the notion that immunological changes occurring within donors of chronic inflammatory conditions do occur, but likely require more extensive analysis methods than we have been able to apply in the current study, including more detailed characterization of condition-related changes in pulmonary immune cells at a transcriptional level.

Collectively, our data suggest that immunological differences that arise during COPD and asthma are not associated with dramatic changes in the overall lung immune cell compartment in those suffering each of these conditions. Instead, they indicate that it is functional changes within key immune cells in these donors that are likely responsible for differing responses to the same stimuli, which may include altered localization of specific cell types, differing metabolic capacities or access to metabolic substrates, different signalling events, or increased exposure to microbial or damage signals.

The data presented here enrich our overall understanding of major immune compartments within the human lung. They demonstrate major phenotypic differences between immune populations in the lung and blood and help establish markers of maturation and activation in the MNP and T cell compartments. Finally, these data suggest that in donors with asthma and COPD, at resting state, the gross composition of the pulmonary immune landscape is comparable to control donors. This indicates that immune dysregulation during these conditions is not a product of changes in the frequency of major immune cell subsets, but rather is likely due to differential functional capacity of these cells upon a sensitization/challenge event or changes in tissue localization. This highlights a need for a shift in phenotypic studies to integrate a focus on cell function and localization, rather than simply surface marker expression.

## Methods and materials

### Donor samples

Lung samples were obtained via the Manchester Allergy, Respiratory and Thoracic Surgery (ManARTs) biobank from donors undergoing resection surgery for suspected lung cancer at Wythenshawe hospital (donors: *n* = 115, median age = 71 year; age range = 28–89 year; 68 female). Written consent was obtained from all donors and the study was approved under the National Research Ethics Service Committee; North West—Haydock ethics reference 20/NW/0302. The research use of the samples was in accord with the terms of the informed consent obtained from the donors. Lung tissue taken forward for processing was a minimum of 40 mm from the tumour site as determined by experienced pathologists and varied from 5 to 25 g in weight. In most cases, samples were processed on the same day as the surgery. Where this was not possible samples were stored at 4^o^C overnight prior to processing. Fresh tissue was deposited in containers prior to transport to prevent drying out. Peripheral blood was collected into EDTA coated tubes and kept a room temperature. Samples were transported to the University of Manchester at ambient temperature.

Asthmatics were self-identified at the point of providing consent (donors: *n* = 17, median age = 72 year, age range = 55–83 year, 14 female). COPD donors were identified through a combination of self-identification, presentation of clinical symptoms including emphysema on chest X-ray, poor lung function measure by spirometry (FEV1/FVC < 70%), and a history of smoking (donors: *n* = 38, median age = 69 year, age range = 45–89 year, 20 female). Control donors were identified as those donors that did not present with asthma or COPD, and did not have a history of other inflammatory conditions, e.g. rheumatoid arthritis (donors: *n* = 60, median age = 71, age range = 28–86, 34 female).

Donors were stratified on smoking status based on self-reported smoking habits. Donors were defined as never smokers if they reported never having smoked, ex-smokers if they reported a history of smoking but reported not having smoked for at least 1 year prior to the date of surgery, or current smokers if they reported having smoked within 1 year prior to the date of surgery.

### Tissue processing

To isolate PBMCs blood was diluted 1:2 in PBS then layered over Ficoll–Paque and centrifuged for 40 min at 400 × g at room temperature. PBMCs were collected with a Pasteur pipette, washed in Lung buffer (PBS + 10% FCS + 2 mM EDTA), counted, and resuspended in freezing medium (10% DMSO in FBS) at ~10–20 million cells/ml. PBMCs were frozen to −150^o^C using a Mr Frosty Freezing Container (ThermoFisher Scientific).

To create single-cell suspensions from lung samples, resected lung tissue was weighed, diced in a sterile petri dish using a sterile razor blade, and transferred into a tube containing digestion buffer (Liberase TL 125 μg/ml, DNase 150 U/ml, in Hanks balanced salt solution H9269). The samples were then incubated at 37^o^C for 40 min in a shaking incubator. Following incubation the samples were passed through a 100 μm filter, 10% FBS Hanks media was added, and then the samples were passed through a 70 μm filter. The samples were then centrifuged (400 × g, 5 min), the supernatant removed and RBC lysis performed with distilled water. Samples were then washed in 10% FBS Hanks media and layered over Ficoll–Paque, and centrifuged for 40 min at 400 × g at room temperature. Mononuclear cells were collected with a Pasteur pipette, washed in lung buffer, counted, and resuspended in freezing medium (10% DMSO in FBS) at ~10–20 million cells/ml. All tissue processing occurred under sterile conditions in a class II safety cabinet. Isolated mononuclear cells were frozen to −150^o^C using a Mr Frosty Freezing Container and stored at −150^o^C until use (ThermoFisher Scientific).

### Flow cytometry

For acquisition of flow cytometric data, PBMCs and mononuclear lung cells were rapidly thawed to room temperature. Cells were incubated with LIVE/DEAD Fixable Blue Dead Cell Stain, then blocked with Human BD Fc block. Antibody staining was performed using the following antibodies: monocyte/DC panel; CD3 (FITC, OKT3), CD19 (FITC, HIB19), CD56 (FITC, HCD56), CD66b (FITC, 3G8), CD1a (PerCP-eF710, HI149), CD172a (APC, SESA5), MHC-II (AF700, LN3), CD14 (APC-eF780, 61D3), CD1c (BV421, L161), CD45 (BV510, HI30), CD11c (BV605, B-ly6), CD86 (BV650, IT2.2), CD141 (BV711, 1A4), CD16 (BV785, 3G8), CD40 (PE, 5C3), CD303 (PE-CF594, 201A), CD206 (PE-Cy7, 15-2).

T cell panel; FOXP3 (FITC, 236A/E7), CD8 (PerCP-ef710, SK8), CD103 (APC, Ber-ACT8), CD4 (AF700, SK3), CD3 (APC-eF780, OKT3), CD45 (BV510, HI30), CD45RA (BV605, HI100), CD69 (BV650, FN50), CD127 (BV711, A019D5), CD28 (PE, CD28.2), TCRγδ (PE-CF594, B1), CCR7 (PE-Cy7, G043H7).

ILC panel; KLRG1 (AF488, 13F12F2), CD117 (PerCP-eF710, 104D2), CD14 (AF700, HCD14), CD11b (AF700, CBRM1/5), CD11c (AF700, 3.9), CD19 (AF700, HIB19), CD127 (APC-eF780, eBioRDR5), CD56 (BV421, HCD56), CD45 (BV605, HI30), HLA-DR (BUV395, G46-6), NKp44 (PE, P44-8), CD3 (PE-Cy7, UCHT1), CD5 (PE-Cy7, UCHT2), FceR1 (PE-Cy7, CRA1).

Other antibodies used; CD11b (PerCP-eF710, ICRF44), CD66b (AF700, 3G8), CD3 (APC-eF780, OKT3), CD45 (BV510, HI30) CD56 (BV650, HCD56), CD16 (BV785, 3G8), Siglec-8 (PE, 7C9), CD19 (PE-Cy5, HIB19), CD64 (PE-Cy7, 10.1).

Intracellular staining for FOXP3 was performed after extracellular staining using the FOXP3/transcription factor staining buffer set. Data was acquired on a BD LSR Fortessa flow cytometer and analysis was conducted on FlowJo V10.8.0.

### Statistical analysis

Statistical analysis was performed using R (R core team 2021) [65] and figures were produced using the ggplot2 package [66]. Unless otherwise stated, statistical values were calculated using unpaired Wilcoxon two-sample tests and adjusted with the Holm method using compare_means in the ggpubr package [67] or rstatix package [68]. Permutational ANOVA was performed using the vegan package [69]. An adjusted *P* value of > 0.05 was considered not statistically significant. Statistical comparisons are denoted either directly above the plot with asterisks or in dedicated supplementary tile plots. *P < 0.05; **P < 0.01; ***P < 0.001; ****P < 0.0001.

## Supplementary Material

kyad009_suppl_Supplementary_Figure_S1

kyad009_suppl_Supplementary_Figure_S2

kyad009_suppl_Supplementary_Figure_S3

kyad009_suppl_Supplementary_Figure_S4

kyad009_suppl_Supplementary_Figure_S5

kyad009_suppl_Supplementary_Figure_S6

kyad009_suppl_Supplementary_Figure_S7

kyad009_suppl_Supplementary_Figure_S8

kyad009_suppl_Supplementary_Figure_S9

kyad009_suppl_Supplementary_Figure_S10

## Data Availability

All data contributing to the conclusions of this manuscript are present in the manuscript and supplementary material. Raw data will be made available upon reasonable request to the corresponding authors.

## Abbreviations

ANOVA: analysis of variance
CCR: C-C chemokine receptor
CD: cluster of differentiation
cDC: conventional dendritic cell
COPD: chronic pulmonary obstructive disease
DC: dendritic cell
DMSO: dimethyl sulfoxide
EDTA: ethylenediaminetetraacetic acid
FBS: fetal bovine serum
FEV: forced expiatory volume
FVC: forced vital capacity
FOXP3: forkhead box P3
IL: interleukin
ILC: innate lymphoid cell
IFN: interferon
ManARTs: Manchester Allergy: Respiratory and Thoracic Surgery biobank
MFI: median fluorescent intensity
MHCII: major histocompatibility complex class II
MNP: mononuclear phagocytes
moDC: monocyte-derived dendritic cell
NCR: natural cytotoxicity receptor
NK: natural killer
NKT: natural killer T cell
PBMC: peripheral blood mononuclear cell
PBS: phosphate buffered saline
PCA: principal coordinates analysis
pDC: plasmacytoid dendritic cell
PMA: phorbol 12-myristate 13-acetate
RBC: red blood cell
SSC-A: side scatter area
SEM: standard error of the mean
SIgA: secretory immunoglobulin A
TCR: T cell receptor
TCM: central memory T cell
TEM: effector memory T cell
TEMRA: T effectors cells re-expressing CD45RA
Th: T helper
Tregs: regulatory T cells
TRM: tissue resident memory T cells

## References

[1] Møller SH , Wang L, Ho PC. Metabolic programming in dendritic cells tailors immune responses and homeostasis. Cell Mol Immunol 2022, 19, 370–83. doi:10.1038/s41423-021-00753-1. 34413487 PMC8891341

[2] Duan W , Croft M. Control of regulatory T Cells and airway tolerance by lung macrophages and dendritic cells. Ann. Am. Thorac. Soc 2014, 11, S306–13. doi:10.1513/AnnalsATS.201401-028AW. 25525738 PMC4298969

[3] Farache J , Zigmond E, Shakhar G, Jung S. Contributions of dendritic cells and macrophages to intestinal homeostasis and immune defense. Immunol Cell Biol 2013, 91, 232–9. doi:10.1038/icb.2012.79. 23399695

[4] Gebhardt T , Palendira U, Tscharke DC, Bedoui S. Tissue-resident memory T cells in tissue homeostasis, persistent infection, and cancer surveillance. Immunol Rev 2018, 283, 54–76. doi:10.1111/imr.12650. 29664571

[5] Sharma A , Rudra D. Emerging functions of regulatory T cells in tissue homeostasis. Front Immunol 2018, 9, 1–26. doi:10.3389/fimmu.2018.00883. 29887862 PMC5989423

[6] Tomlin H , Piccinini AM. A complex interplay between the extracellular matrix and the innate immune response to microbial pathogens. Immunology 2018, 155, 186–201. doi:10.1111/imm.12972. 29908065 PMC6142291

[7] Elmentaite R , Teichmann SA, Madissoon E. Studying immune to nonimmune cell cross-talk using single-cell technologies. Curr Opin Syst Biol 2019, 18, 87–94. doi:10.1016/j.coisb.2019.10.005. 32984660 PMC7493433

[8] Hu W , Pasare C. Location, location, location: tissue-specific regulation of immune responses. J Leukoc Biol 2013, 94, 409–21. doi:10.1189/jlb.0413207. 23825388 PMC3747123

[9] Mann ER , Menon M, Knight SB, Konkel JE, Jagger C, Shaw TN, et al. Longitudinal immune profiling reveals key myeloid signatures associated with COVID-19. Sci Immunol 2020, 5, 1–15. doi:10.1126/sciimmunol.abd6197. PMC7857390 32943497

[10] Mathew D , Giles JR, Baxter AE, Oldridge DA, Greenplate AR, Wu JE, et al.; UPenn COVID Processing Unit. Deep immune profiling of COVID-19 patients reveals distinct immunotypes with therapeutic implications. Science (80-.) 2020, 369, eabc8511. doi:10.1126/science.abc8511. PMC7402624 32669297

[11] Pallett LJ , Davies J, Colbeck EJ, Robertson F, Hansi N, Easom NJW, et al. IL-2 high tissue-resident T cells in the human liver: Sentinels for hepatotropic infection. J Exp Med 2017, 214, 1567–80. doi:10.1084/jem.20162115. 28526759 PMC5461007

[12] Smolders J , Heutinck KM, Fransen NL, Remmerswaal EBM, Hombrink P, ten Berge IJM, et al. Tissue-resident memory T cells populate the human brain. Nat Commun 2018, 9, 1–14. doi:10.1038/s41467-018-07053-9. 30389931 PMC6214977

[13] Baharom F , Rankin G, Blomberg A, Smed-Sörensen A. Human lung mononuclear phagocytes in health and disease. Front Immunol 2017, 8, 1–16. doi:10.3389/fimmu.2017.00499. 28507549 PMC5410584

[14] Lambrecht BN , Hammad H. Asthma: The importance of dysregulated barrier immunity. Eur J Immunol 2013, 43, 3125–37. doi:10.1002/eji.201343730. 24165907

[15] Wilkinson TMA. Immune checkpoints in chronic obstructive pulmonary disease. Eur Respir Rev 2017, 26, 170045. doi:10.1183/16000617.0045-2017. 28659497 PMC9489040

[16] Merad M , Sathe P, Helft J, Miller J, Mortha A. The dendritic cell lineage: Ontogeny and function of dendritic cells and their subsets in the steady state and the inflamed setting. Annu Rev Immunol 2013, 31, 563–604. doi:10.1146/annurev-immunol-020711-074950. 23516985 PMC3853342

[17] Dutertre CA , Becht E, Irac SE, Khalilnezhad A, Narang V, Khalilnezhad S, et al. Single-cell analysis of human mononuclear phagocytes reveals subset-defining markers and identifies circulating inflammatory dendritic cells. Immunity 2019, 51, 573–589.e8. doi:10.1016/j.immuni.2019.08.008. 31474513

[18] Angkasekwinai P , Park H, Wang Y-H, Wang Y-H, Chang SH, Corry DB, et al. Interleukin 25 promotes the initiation of proallergic type 2 responses. J Exp Med 2007, 204, 1509–17. doi:10.1084/jem.20061675. 17562814 PMC2118650

[19] See P , Dutertre C-A, Chen J, Günther P, McGovern N, Irac SE, et al. Mapping the human DC lineage through the integration of high-dimensional techniques. Science (80-.) 2017, 356, eaag3009. doi:10.1126/science.aag3009. PMC7611082 28473638

[20] Alcántara-Hernández M , Leylek R, Wagar LE, Engleman EG, Keler T, Marinkovich MP, et al. High-dimensional phenotypic mapping of human dendritic cells reveals interindividual variation and tissue specialization. Immunity 2017, 47, 1037–1050.e6. doi:10.1016/j.immuni.2017.11.001. 29221729 PMC5738280

[21] Guilliams M , Dutertre C-A, Scott Charlotte L, McGovern N, Sichien D, Chakarov S, et al. Unsupervised high-dimensional analysis aligns dendritic cells across tissues and species. Immunity 2016, 45, 669–84. doi:10.1016/j.immuni.2016.08.015. 27637149 PMC5040826

[22] Granot T , Senda T, Carpenter DJ, Matsuoka N, Weiner J, Gordon Claire L, et al. Dendritic cells display subset and tissue-specific maturation dynamics over human life. Immunity 2017, 46, 504–15. doi:10.1016/j.immuni.2017.02.019. 28329707 PMC5415308

[23] Upham JW , Xi Y. Dendritic cells in human lung disease: recent advances. Chest 2017, 151, 668–73. doi:10.1016/j.chest.2016.09.030. 27729261

[24] Snyder ME , Farber DL. Human lung tissue resident memory T cells in health and disease. Curr Opin Immunol 2019, 59, 101–8. doi:10.1016/j.coi.2019.05.011. 31265968 PMC6774897

[25] Chen K , Kolls JK. T cell-mediated host immune defenses in the lung. Annu Rev Immunol 2013, 31, 605–33. doi:10.1146/annurev-immunol-032712-100019. 23516986 PMC3912562

[26] Panduro M , Benoist C, Mathis D. Tissue Tregs. Annu Rev Immunol 2016, 34, 609–33. doi:10.1146/annurev-immunol-032712-095948. 27168246 PMC4942112

[27] Arpaia N , Green JA, Moltedo B, Arvey A, Hemmers S, Yuan S, et al. A distinct function of regulatory T cells in tissue protection. Cell 2015, 162, 1078–89. doi:10.1016/j.cell.2015.08.021. 26317471 PMC4603556

[28] Ribot JC , Lopes N, Silva-Santos B. γδ T cells in tissue physiology and surveillance. Nat Rev Immunol 2021, 21, 221–32. doi:10.1038/s41577-020-00452-4. 33057185

[29] Kumar BV , Ma W, Miron M, Granot T, Guyer RS, Carpenter DJ, et al. Human tissue-resident memory T cells are defined by core transcriptional and functional signatures in lymphoid and mucosal sites. Cell Rep 2017, 20, 2921–34. doi:10.1016/j.celrep.2017.08.078. 28930685 PMC5646692

[30] Schreurs RRCE , Sagebiel AF, Steinert FL, Highton AJ, Klarenbeek PL, Drewniak A, et al. Intestinal CD8+ T cell responses are abundantly induced early in human development but show impaired cytotoxic effector capacities. Mucosal Immunol 2021, 14, 605–14. doi:10.1038/s41385-021-00382-x. 33772147 PMC8075922

[31] Pallett LJ , Burton AR, Amin OE, Rodriguez-Tajes S, Patel AA, Zakeri N, et al. Longevity and replenishment of human liver-resident memory T cells and mononuclear phagocytes. J Exp Med 2020, 217, 2457–8. doi:10.1084/jem.20200050. PMC7478732 32602903

[32] Woodward Davis AS , Vick Sarah C, Pattacini L, Voillet V, Hughes SM, Lentz GM, et al. The human memory T cell compartment changes across tissues of the female reproductive tract. Mucosal Immunol 2021, 14, 862–72. doi:10.1038/s41385-021-00406-6. 33953338 PMC8225572

[33] Sathaliyawala T , Kubota M, Yudanin N, Turner D, Camp P, Thome Joseph JC, et al. Distribution and compartmentalization of human circulating and tissue-resident memory T cell subsets. Immunity 2013, 38, 187–97. doi:10.1016/j.immuni.2012.09.020. 23260195 PMC3557604

[34] Thome JJC , Yudanin N, Ohmura Y, Kubota M, Grinshpun B, Sathaliyawala T, et al. Spatial map of human T cell compartmentalization and maintenance over decades of life. Cell 2014, 159, 814–28. doi:10.1016/j.cell.2014.10.026. 25417158 PMC4243051

[35] Wong MT , Ong DEH, Lim FSH, Teng KWW, McGovern N, Narayanan S, et al. A high-dimensional atlas of human T cell diversity reveals tissue-specific trafficking and cytokine signatures. Immunity 2016, 45, 442–56. doi:10.1016/j.immuni.2016.07.007. 27521270

[36] Guilliams M , Ginhoux F, Jakubzick C, Naik SH, Onai N, Schraml BU, et al. Dendritic cells, monocytes and macrophages: a unified nomenclature based on ontogeny. Nat Rev Immunol 2014, 14, 571–8. doi:10.1038/nri3712. 25033907 PMC4638219

[37] Collin M , Bigley V. Human dendritic cell subsets: an update. Immunology 2018, 154, 3–20. doi:10.1111/imm.12888. 29313948 PMC5904714

[38] Gordon S. Alternative activation of macrophages. Nat Rev Immunol 2003, 3, 23–35. doi:10.1038/nri978. 12511873

[39] Thome JJC , Bickham Kara L, Ohmura Y, Kubota M, Matsuoka N, Gordon C, et al. Early-life compartmentalization of human T cell differentiation and regulatory function in mucosal and lymphoid tissues. Nat Med 2016, 22, 72–7. doi:10.1038/nm.4008. 26657141 PMC4703455

[40] Shenderov K , Collins SL, Powell JD, Horton MR. Immune dysregulation as a driver of idiopathic pulmonary fibrosis. J Clin Invest 2021, 131, 1–9. doi:10.1172/JCI143226. PMC7810481 33463535

[41] McKenzie ANJ , Spits H, Eberl G. Innate lymphoid cells in inflammation and immunity. Immunity 2014, 41, 366–74. doi:10.1016/j.immuni.2014.09.006. 25238094

[42] Huang C , Li F, Wang J, Tian Z. Innate-like lymphocytes and innate lymphoid cells in asthma. Clin Rev Allergy Immunol 2020, 59, 359–70. doi:10.1007/s12016-019-08773-6. 31776937

[43] Blomme EE , Provoost S, De Smet EG, De Grove Katrien C, Van Eeckhoutte HP, De Volder J, et al. Quantification and role of innate lymphoid cell subsets in chronic obstructive pulmonary disease. Clin Transl Immunol 2021, 10, 1–16. doi:10.1002/cti2.1287. PMC8178740 34136217

[44] Silver JS , Kearley J, Copenhaver AM, Sanden C, Mori M, Yu L, et al. Inflammatory triggers associated with exacerbations of COPD orchestrate plasticity of group 2 innate lymphoid cells in the lungs. Nat Immunol 2016, 17, 626–35. doi:10.1038/ni.3443. 27111143 PMC5345745

[45] Grove KCD , et al. Characterization and quantification of innate lymphoid cell subsets in human lung. PLoS One 2016, 11, 1–12. doi:10.1371/journal.pone.0145961. PMC4699688 26727464

[46] Naessens T , Morias Y, Hamrud E, Gehrmann U, Budida R, Mattsson J, et al. Human lung conventional dendritic cells orchestrate lymphoid Neogenesis during chronic obstructive pulmonary disease. Am J Respir Crit Care Med 2020, 202, 535–48. doi:10.1164/rccm.201906-1123OC. 32255375 PMC7616955

[47] Domínguez Conde C , Xu C, Jarvis LB, Rainbow DB, Wells SB, Gomes T, et al. Cross-tissue immune cell analysis reveals tissue-specific features in humans. Science (80-.) 2022, 376, eabl5197. doi:10.1126/science.abl5197. PMC7612735 35549406

[48] Desch AN , Gibbings Sophie L, Goyal R, Kolde R, Bednarek J, Bruno T, et al. Flow cytometric analysis of mononuclear phagocytes in nondiseased human lung and lung-draining lymph nodes. Am J Respir Crit Care Med 2016, 193, 614–26. doi:10.1164/rccm.201507-1376OC. 26551758 PMC4824940

[49] Kerrigan AM , Brown GD. C-type lectins and phagocytosis. Immunobiology 2009, 214, 562–75. doi:10.1016/j.imbio.2008.11.003. 19261355 PMC2702671

[50] Cochand L , Isler P, Songeon F, Nicod LP. Human lung dendritic cells have an immature phenotype with efficient mannose receptors. Am J Respir Cell Mol Biol 1999, 21, 547–54. doi:10.1165/ajrcmb.21.5.3785. 10536111

[51] Oja AE , Piet B, Helbig C, Stark R, van der Zwan D, Blaauwgeers H, et al. Trigger-happy resident memory CD4 + T cells inhabit the human lungs. Mucosal Immunol 2018, 11, 654–67. doi:10.1038/mi.2017.94. 29139478

[52] Snyder ME , Finlayson MO, Connors TJ, Dogra P, Senda T, Bush E, et al. Generation and persistence of human tissue-resident memory T cells in lung transplantation. Sci Immunol 2019, 4, eaav5581. doi:10.1126/sciimmunol.aav5581. 30850393 PMC6435356

[53] Hombrink P , Helbig C, Backer RA, Piet B, Oja AE, Stark R, et al. Programs for the persistence, vigilance and control of human CD8 + lung-resident memory T cells. Nat Immunol 2016, 17, 1467–78. doi:10.1038/ni.3589. 27776108

[54] Lambrecht BN , Hammad H. The immunology of asthma. Nat Immunol 2015, 16, 45–56. doi:10.1038/ni.3049. 25521684

[55] Wouters EFM , Reynaert NL, Dentener MA, Vernooy JHJ. Systemic and local inflammation in asthma and chronic obstructive pulmonary disease is there a connection? Proc Am Thorac Soc 2009, 6, 638–47. doi:10.1513/pats.200907-073DP. 20008867

[56] Richmond BW , Mansouri S, Serezani A, Novitskiy S, Blackburn JB, Du R-H, et al. Monocyte-derived dendritic cells link localized secretory IgA deficiency to adaptive immune activation in COPD. Mucosal Immunol 2021, 14, 431–42. doi:10.1038/s41385-020-00344-9. 32968197 PMC7946625

[57] Pons J , Sauleda J, Ferrer JM, Barceló B, Fuster A, Regueiro V, et al. Blunted γδ T-lymphocyte response in chronic obstructive pulmonary disease. Eur Respir J 2005, 25, 441–6. doi:10.1183/09031936.05.00069304. 15738286

[58] Panda SK , Colonna M. Innate lymphoid cells in mucosal immunity. Front Immunol 2019, 10, 1–13. doi:10.3389/fimmu.2019.00861. 31134050 PMC6515929

[59] Carrega P , Loiacono F, Di Carlo E, Scaramuccia A, Mora M, Conte R, et al. NCR+ ILC3 concentrate in human lung cancer and associate with intratumoral lymphoid structures. Nat Commun 2015, 6, 8280. doi:10.1038/ncomms9280. 26395069

[60] Hayashi Y , Ishii Y, Hata-Suzuki M, Arai R, Chibana K, Takemasa A, et al. Comparative analysis of circulating dendritic cell subsets in patients with atopic diseases and sarcoidosis. Respir Res 2013, 14, 1–8. doi:10.1186/1465-9921-14-29. 23497225 PMC3599330

[61] Dua B , Watson RM, Gauvreau GM, O’Byrne PM. Myeloid and plasmacytoid dendritic cells in induced sputum after allergen inhalation in subjects with asthma. J Allergy Clin Immunol 2010, 126, 133–9. doi:10.1016/j.jaci.2010.04.006. 20538329

[62] El-Gammal A , Oliveria J-P, Howie K, Watson R, Mitchell P, Chen R, et al. Allergen-induced changes in bone marrow and airway dendritic cells in subjects with asthma. Am J Respir Crit Care Med 2016, 194, 169–77. doi:10.1164/rccm.201508-1623OC. 26844926

[63] Greer AM , Matthay MA, Kukreja J, Bhakta NR, Nguyen CP, Wolters PJ, et al. Accumulation of BDCA1+ dendritic cells in interstitial fibrotic lung diseases and Th2-high asthma. PLoS One 2014, 9, e99084. doi:10.1371/journal.pone.0099084. 24915147 PMC4051692

[64] Vieira Braga FA , Kar G, Berg M, Carpaij OA, Polanski K, Simon LM, et al. A cellular census of human lungs identifies novel cell states in health and in asthma. Nat Med 2019, 25, 1153–63. doi:10.1038/s41591-019-0468-5. 31209336

[65] R Core Team. R: A Language and Environment for Statistical Computing. R Foundation for Statistical Computing, Vienna, Austria. https://www.r-project.org/, 2022.

[66] Wickham, H. ggplot2: Elegant Graphics for Data Analysis. New York: Springer-Verlag. <https://ggplot2.tidyverse.org>, 2016.

[67] Kassambara, A. ggpubr: 'ggplot2' Based Publication Ready Plots. R package version 0.6.0. <https://rpkgs.datanovia.com/ggpubr/>, 2023.

[68] Kassambara, A. rstatix: Pipe-Friendly Framework for Basic Statistical Tests. 2021.

[69] Oksanen, J, Blanchet FG, Friendly M, Kindt R, Legendre P, McGlinn D, et al. vegan: Community Ecology Package. R package version 2.5-7. <https://cran.r-project.org/package=vegan>, 2020.

