# Supplementary figures and images for: Comparative phenotype of circulating versus tissue immune cells in human lung and blood compartments during health and disease
Source: Discov Immunol. 2023 Jul 19;2(1):kyad009. doi: 10.1093/discim/kyad009 (PMC10403752; doi:10.1093/discim/kyad009)

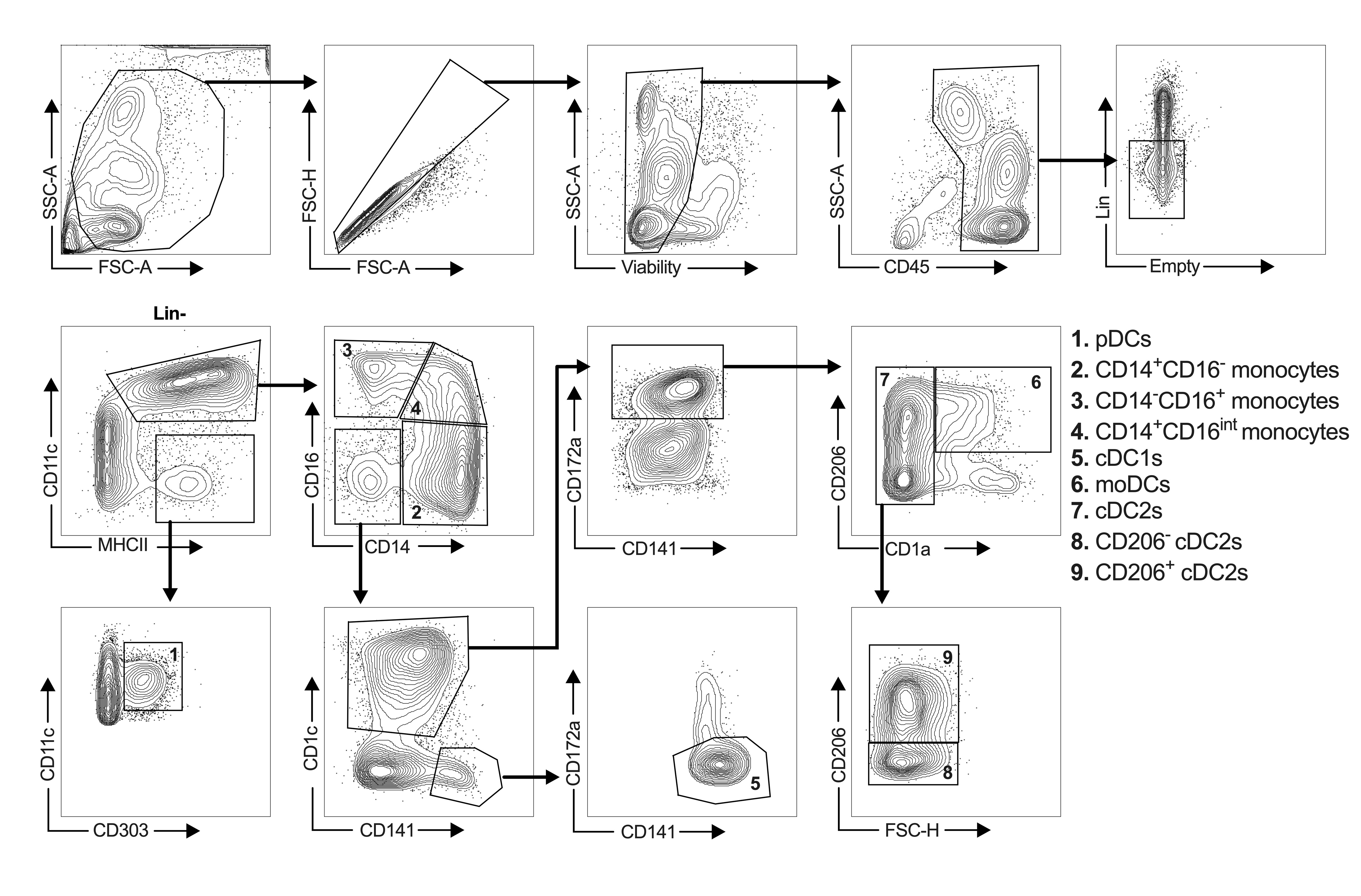

Supplement: kyad009_suppl_Supplementary_Figure_S1 [file kyad009_suppl_Supplementary_Figure_S1.jpg]

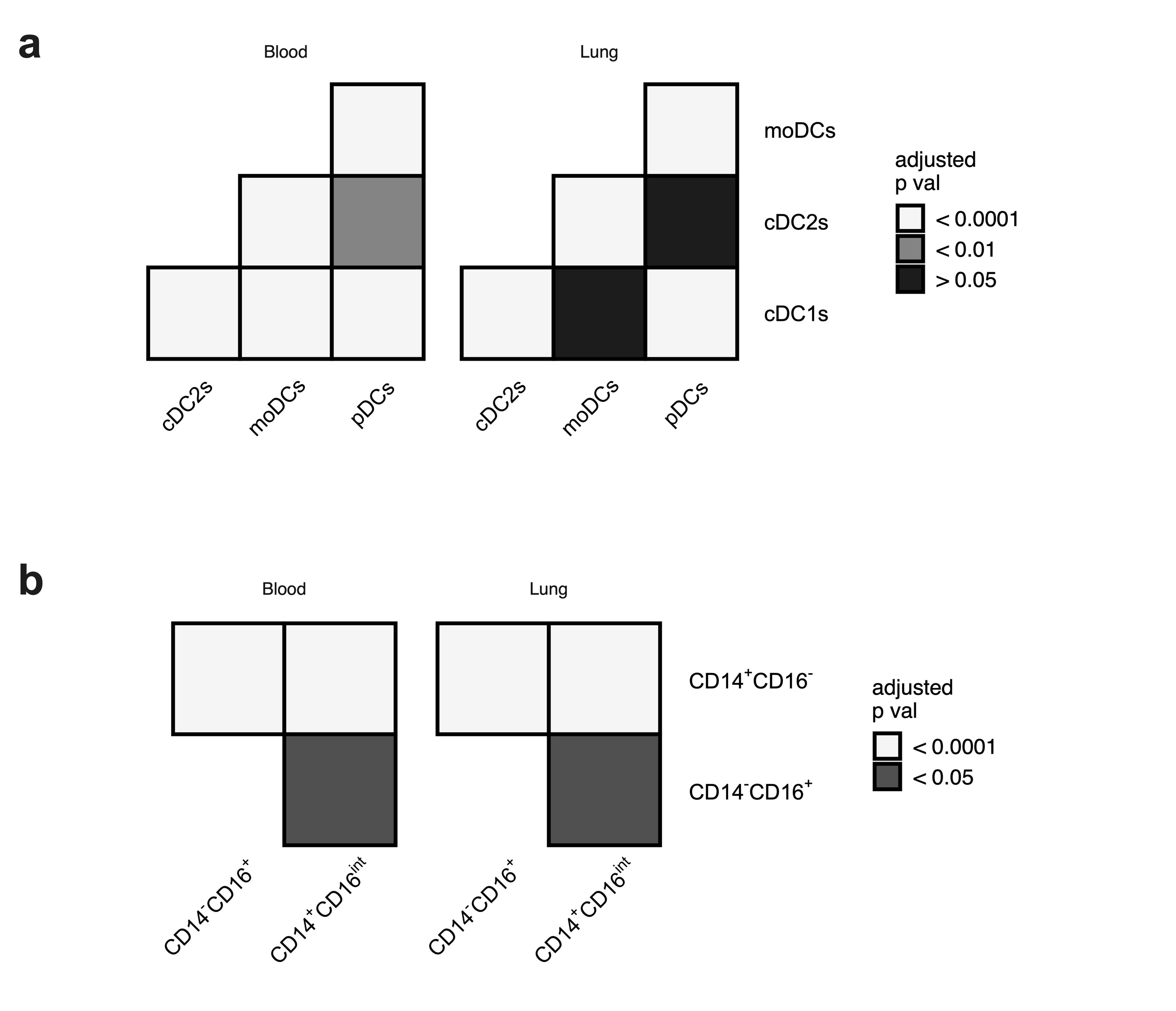

Supplement: kyad009_suppl_Supplementary_Figure_S2 [file kyad009_suppl_Supplementary_Figure_S2.jpg]

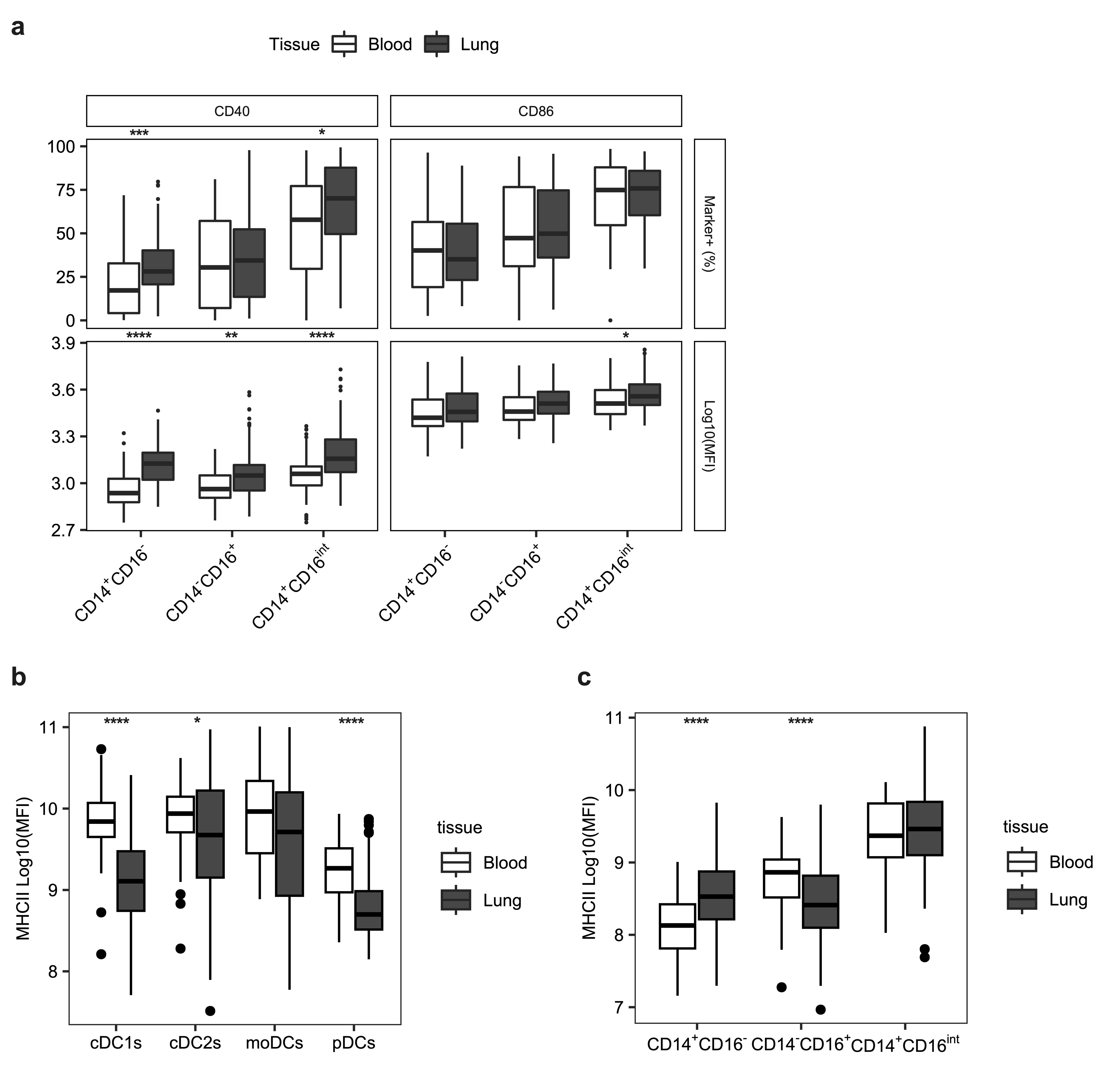

Supplement: kyad009_suppl_Supplementary_Figure_S3 [file kyad009_suppl_Supplementary_Figure_S3.jpg]

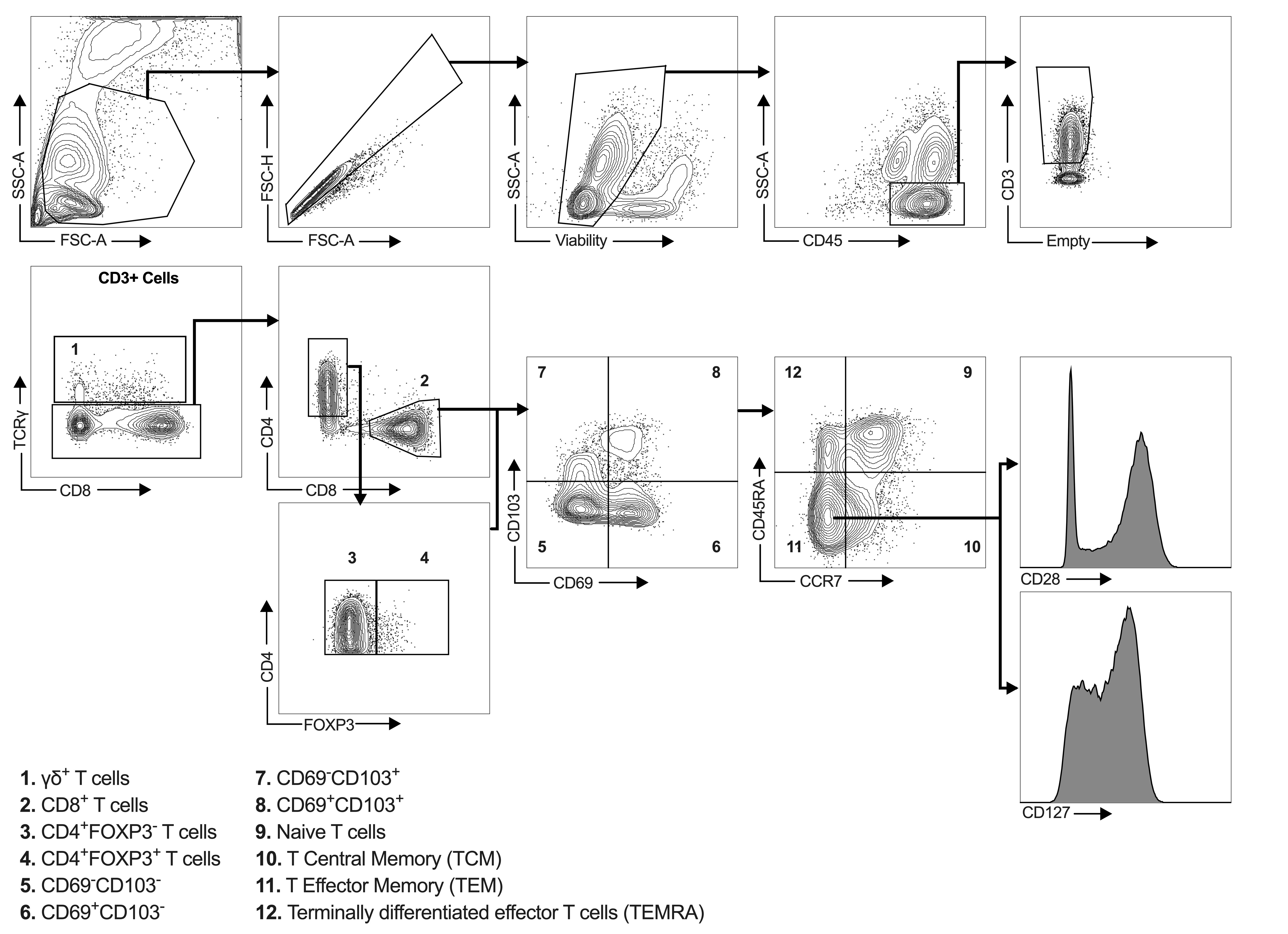

Supplement: kyad009_suppl_Supplementary_Figure_S4 [file kyad009_suppl_Supplementary_Figure_S4.jpg]

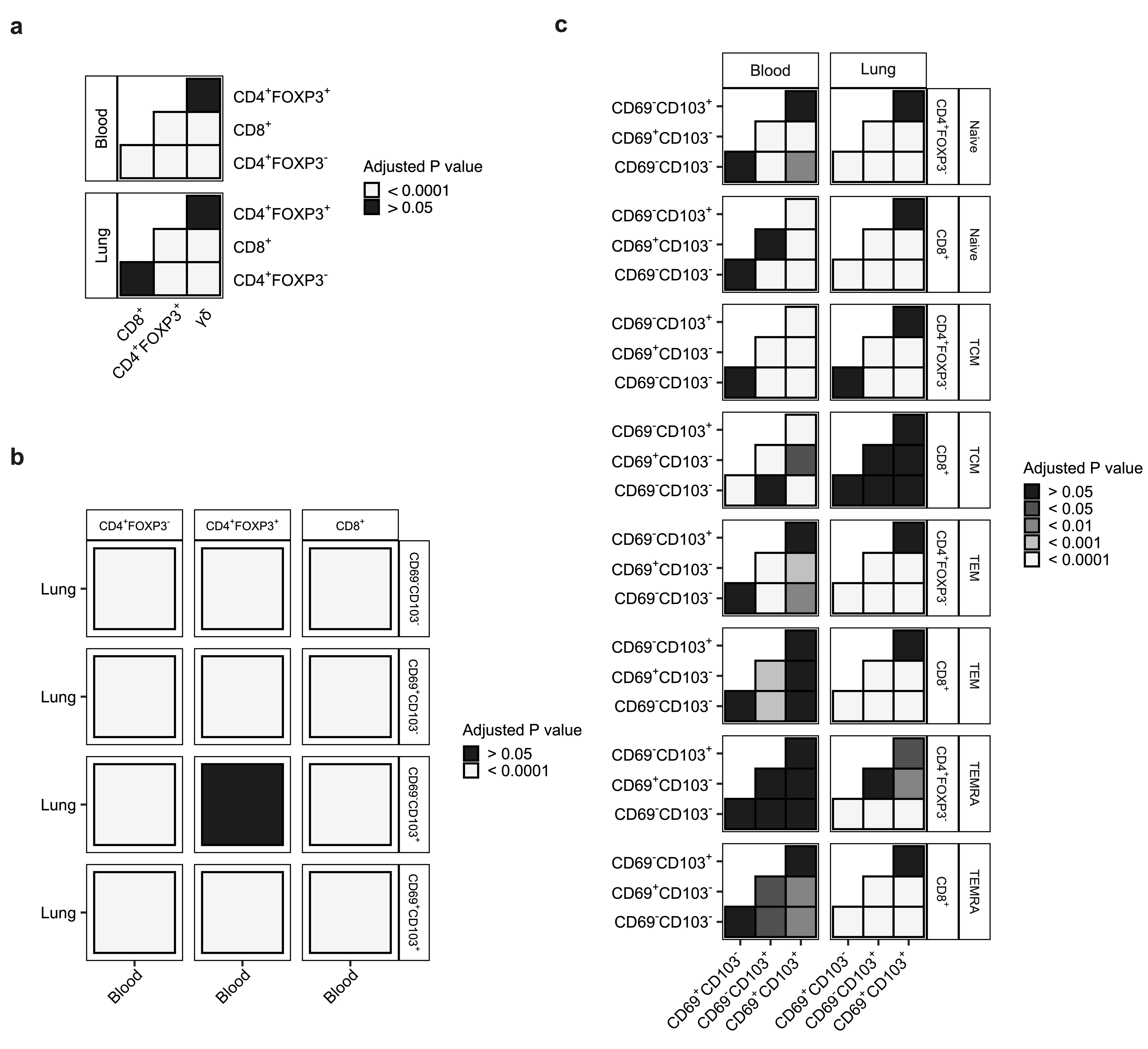

Supplement: kyad009_suppl_Supplementary_Figure_S5 [file kyad009_suppl_Supplementary_Figure_S5.jpg]

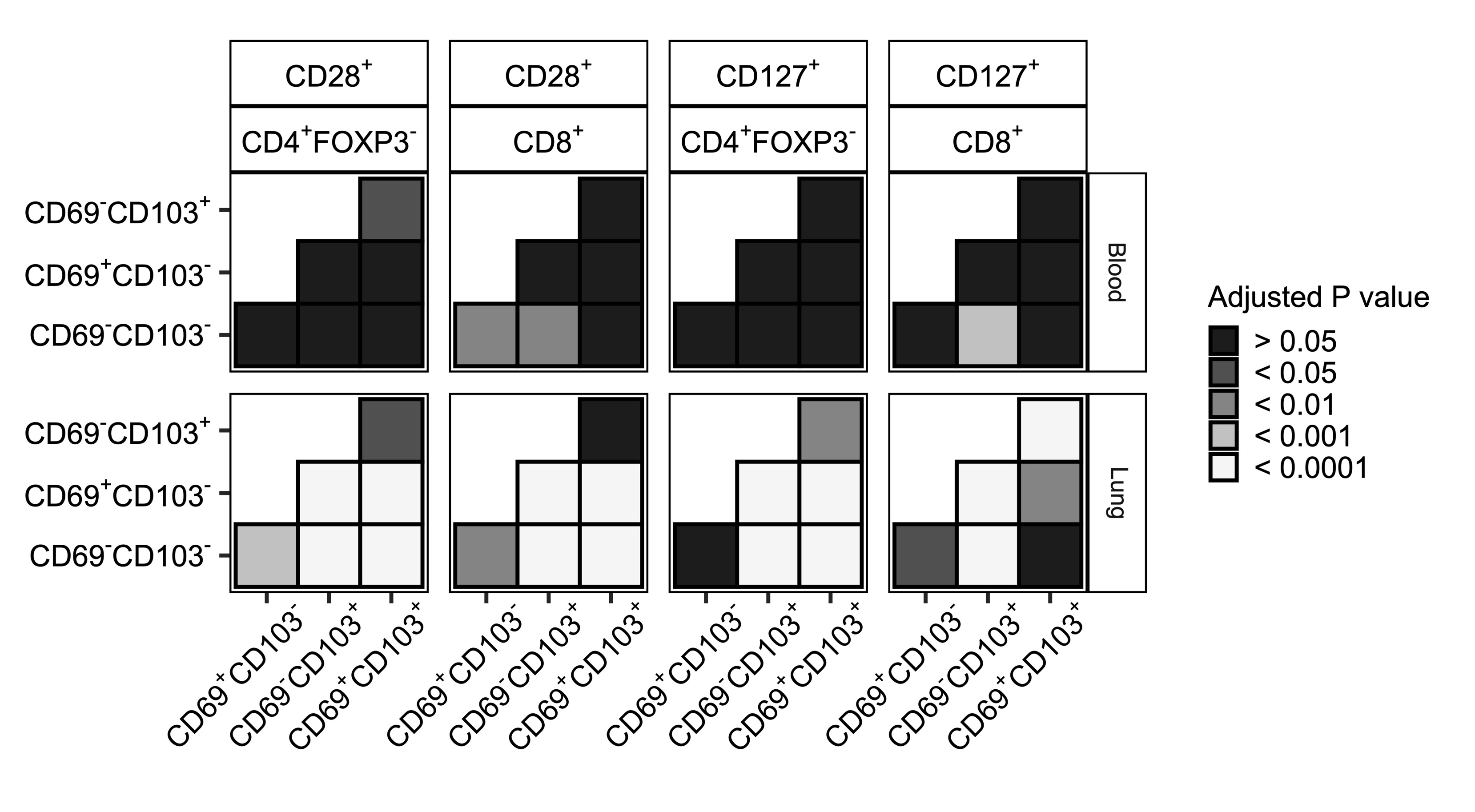

Supplement: kyad009_suppl_Supplementary_Figure_S6 [file kyad009_suppl_Supplementary_Figure_S6.jpg]

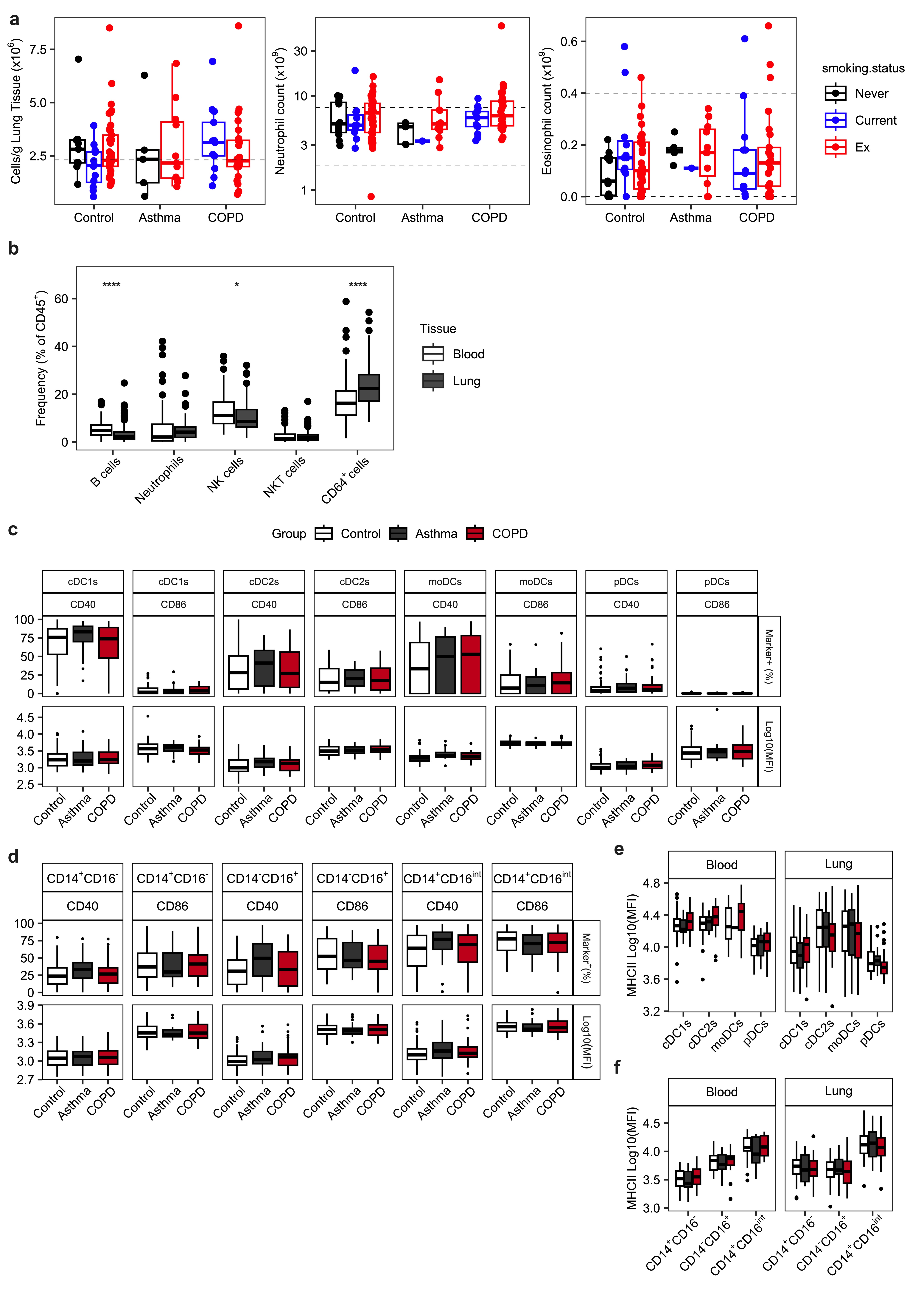

Supplement: kyad009_suppl_Supplementary_Figure_S7 [file kyad009_suppl_Supplementary_Figure_S7.jpg]

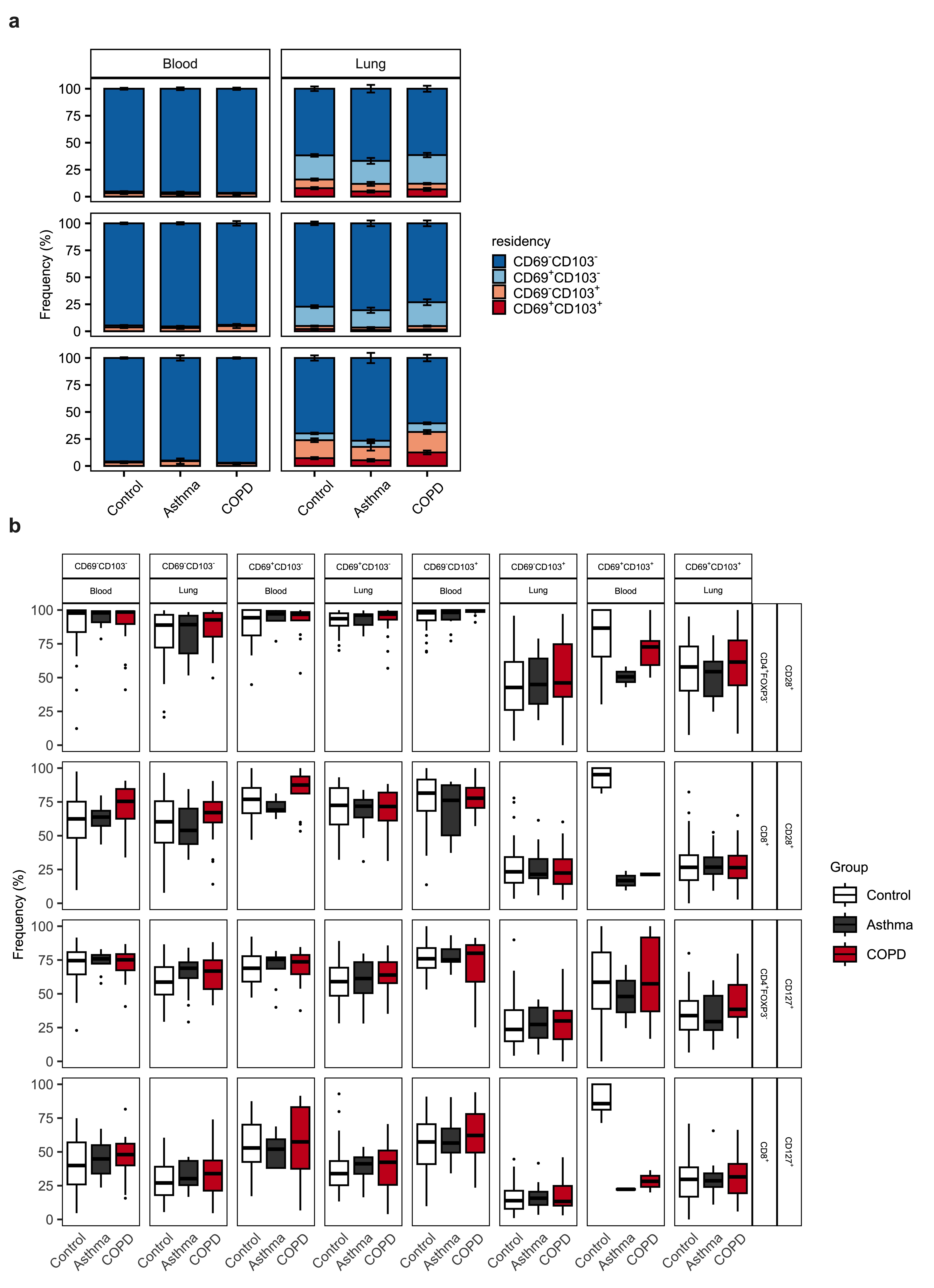

Supplement: kyad009_suppl_Supplementary_Figure_S8 [file kyad009_suppl_Supplementary_Figure_S8.jpg]

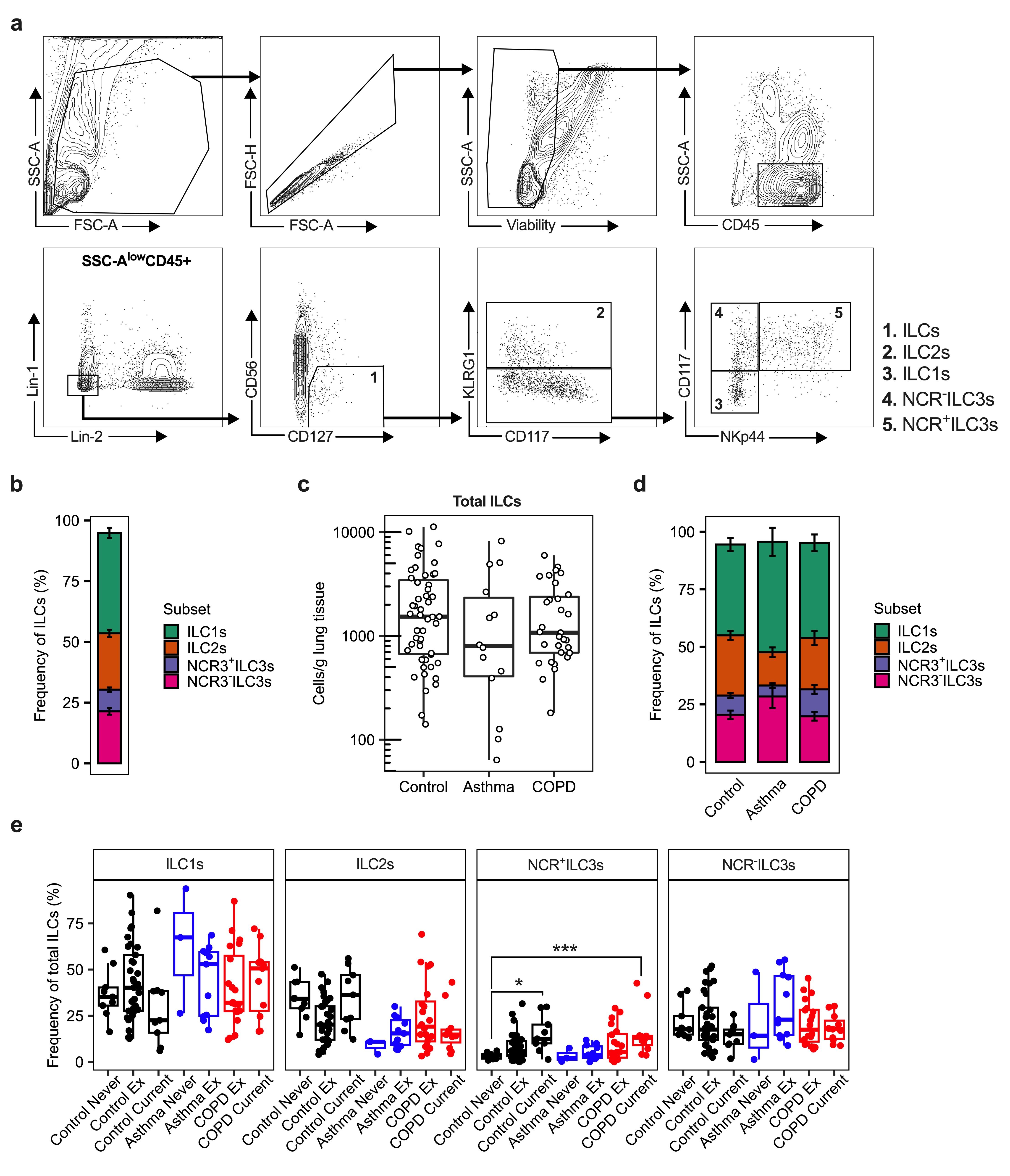

Supplement: kyad009_suppl_Supplementary_Figure_S9 [file kyad009_suppl_Supplementary_Figure_S9.jpg]

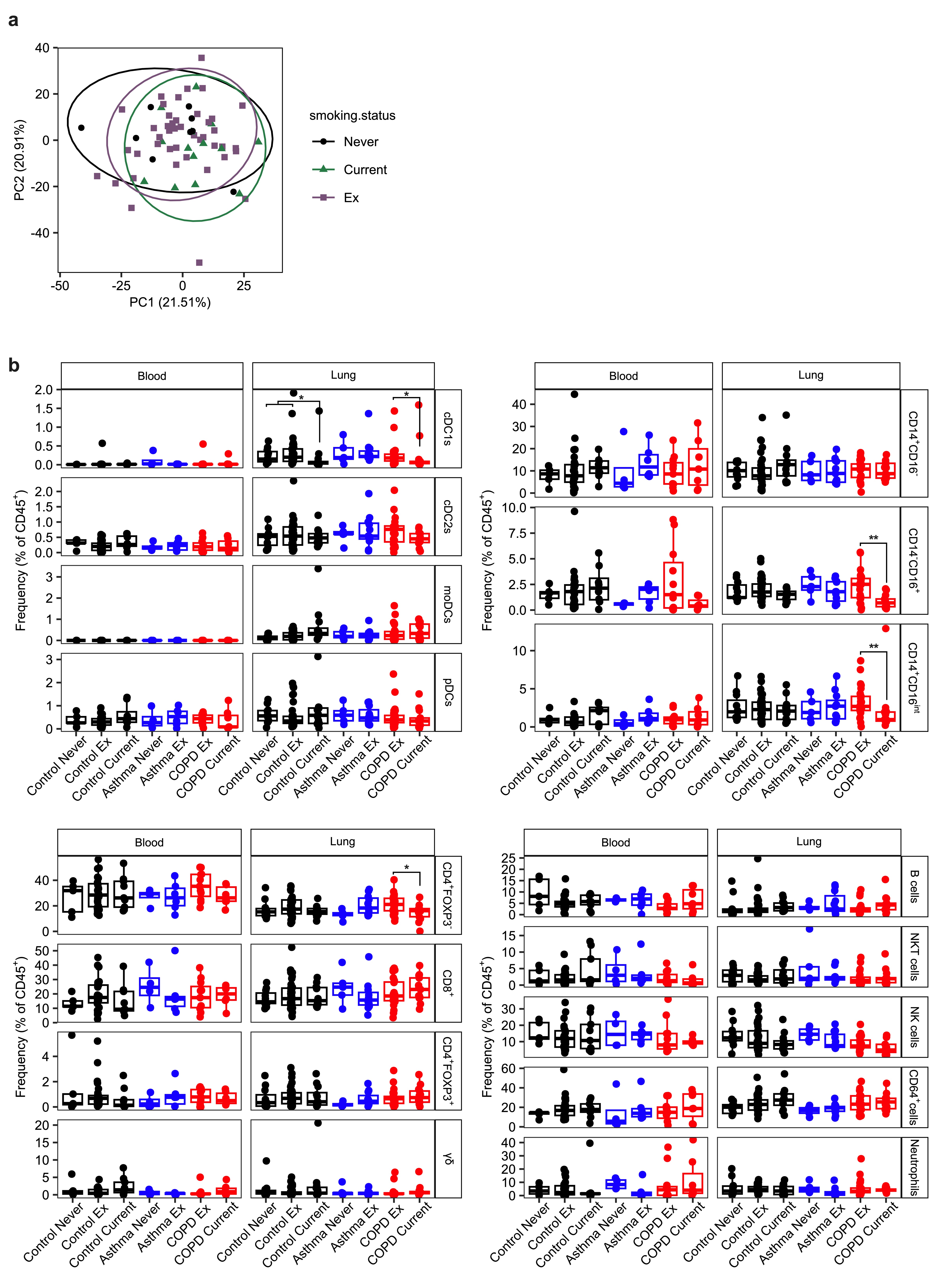

Supplement: kyad009_suppl_Supplementary_Figure_S10 [file kyad009_suppl_Supplementary_Figure_S10.jpg]
